# Synthesis of dehydrodipeptide esters and their evaluation as inhibitors of cathepsin C

**DOI:** 10.1007/s00044-015-1366-0

**Published:** 2015-04-16

**Authors:** Maciej Makowski, Paweł Lenartowicz, Bartosz Oszywa, Michał Jewgiński, Małgorzata Pawełczak, Paweł Kafarski

**Affiliations:** Faculty of Chemistry, Opole University, Oleska 48, 45-052 Opole, Poland; Department of Bioorganic Chemistry, Faculty of Chemistry, Wroclaw University of Technology, Wybrzeze Wyspianskiego 27, 50-370 Wroclaw, Poland

**Keywords:** Dehydropeptides, Esterification, Enzyme inhibitors, Molecular modeling

## Abstract

The procedures for the synthesis of esters of dehydropeptides containing C-terminal (Z)-dehydrophenylalanine and dehydroalanine have been elaborated. These esters appeared to be moderate or weak inhibitors of cathepsin C, with some of them exhibiting slow-binding behavior. As shown by molecular modeling, they are rather bound at the surface of the enzyme and are not submersed in its binding cavities.

**Electronic supplementary material:**

The online version of this article (doi:10.1007/s00044-015-1366-0) contains supplementary material, which is available to authorized users.

## Introduction

*α,β*-Dehydroaminoacids present in proteins contribute to catalytic action in tyrosine aminomutase (Christenson *et al.,*^2003^) and to properties of green fluorescent proteins (Zimmer, 2002). They are also constituents of a variety of peptidic allelochemicals of microbial origin, including antimicrobial lantibiotics (nisin, subtilin, epidermin and gallidermin) (Willey and van der Donk, 2007), neurotoxins (roquefortine, oxaline and phomopsins) (Overy *et al.*, 2005; Battilani *et al.*, 2011), hepatotoxins (microcystins and nodularins) (Gulledge *et al.*, 2002), phytotoxins (tentoxin and AM toxins) (Andre and Pinet, 1997; Jingfeng *et al.*, 2013) and antitumor agents (phenylahistin) (Kanoh *et al.*, 1999). This is because of both, the reactivity of their side-chain double bonds (especially toward thiols) (Ferreira *et al.*, 2001; Seebeck *et al.*, 2011) and of the ability to undertake specific forms of three-dimensional structure [they could be considered as foldamers (Goldman *et al.*, 2007)]. The latter properties cause the growing interest in this class of compounds.

Although from some years we have been engaged in studies on the dependence of three-dimensional structure of dehydropeptides on their inhibitory activity toward cathepsin C, no clear structure–activity relationship could be drawn (Makowski *et al.*, 2001; Latajka *et al.*, 2006, 2008). In this paper, we present synthesis of esters of glycyl^Z^dehydrophenylalanine (Gly-^Z^ΔPhe), glycyldehydroalanine (Gly-ΔAla) and l-phenylalanyldehydroalanine (Phe-ΔAla) and evaluation of their action toward this enzyme.

## Materials and methods

### General

All reagents and solvents were purchased from Sigma-Aldrich, Avantor Performance Materials or Merck. Ethyl acetate (EtOAc), dichloromethane (DCM), diethyl ether (Et_2_O) and tetrahydrofuran (THF) were dried over P_2_O_5_ and then distilled. *N*,*N*-dimethyformamide (DMF) was distilled under reduced pressure and stored over molecular sieves 4 Å. Other chemicals were used without purification. Reaction progress was monitored by TLC on Merck 60 silica plates. The spots were visualized by placing chromatogram plate at chlorine vapor followed by spraying with *o*-tolidine in water/acetic acid mixture. NMR spectra were recorded on Bruker Ultrashield 400 MHz instrument, operating at 400 MHz (^1^H) and 100 MHz (^13^C). Samples were prepared in DMSO-d6 (99.8 % at. D). Chemical shifts are reported in ppm relative to TMS used as internal standard or to the signal of solvent (^1^H NMR 2.5 ppm; ^13^C NMR 39.52 ppm for DMSO-d6), and coupling constant is reported in Hertz. In the description of dipeptide ^1^H NMR and ^13^C NMR spectra, the tosylate group is omitted for better readability (Tos ^1^H NMR (DMSO, 400 MHz): δ 7.48 (d, *J* = 8.0 Hz, 2H, Ar***H***), 7.11 (d, *J* = 8.0 Hz, 2H, Ar***H***), 2.29 (s, 3H, C***H***_3_); ^13^C NMR (DMSO, 100 MHz): δ 20.84 (***C***H_3_), 125.57, 128.14, 137.80, 145.71 (4 × Ar***C***). The copies of all NMR spectra are available at electronic supplementary material. Melting points were determined on a Stuart SMP30 apparatus and are reported uncorrected. Mass spectra were recorded on Bruker micrOTOF-Q II high-resolution mass spectrometer with electrospray ionization (ESI). IR spectra were recorded on Nicolet 6700 FT-IR spectrophotometer (Thermo Scientific) operating at resolution 2 cm^−1^ and scanning range 4000–400 cm^−1^. Samples were measured as KBr disks.

### Synthesis of *N*-protected dehydrodipeptides

Boc-protected dehydrodipeptides containing C-terminal dehydroalanine (ΔAla) or (Z)-dehydrophenylalanine (Δ^Z^Phe) were synthesized earlier by condensation of appropriate carboxamides with α-keto acids in benzene in the presence of *p*-toluenesulfonic acid as catalyst (Makowski *et al.*, 1985).

### Synthesis of dehydrodipeptide methyl, ethyl and isopropyl esters

Syntheses were based on procedure of Cossec *et al.* (2008). Thus, Boc-Gly-ΔAla or Boc-(*S*)Phe-ΔAla was dissolved in methanol (0.2 or 0.4 M, respectively), and 0.5 equivalent of Cs_2_CO_3_ was added. The mixture was stirred for 1 h at room temperature followed by evaporation of solvent. The dipeptide cesium salt was dissolved in DMF (0.28 M), and fivefold or fourfold excess (respectively) of methyl, ethyl or *iso*propyl iodide was added in portions. After completion of the reaction (3–5 h, controlled by TLC), solvent was evaporated under reduced pressure. The obtained residue was dissolved in ethyl acetate and washed subsequently with: 1 M HCl, saturated KHCO_3_, 0.1 M Na_2_S_2_O_3_ and brine (each one in triplicate). Organic layer was dried over anhydrous MgSO_4_. Product was crystallized from mixtures of diethyl ether/hexane or ethyl acetate/hexane providing Boc-Gly-ΔAla-OMe in 91 %, Boc-(S)Phe-ΔAla-OMe in 94 %, Boc-(S)Phe-ΔAla-OEt in 94 % and Boc-(S)Phe-ΔAla-OPr^i^ in 81 % yields. Deprotection of amine group was performed in 20 % solution of TFA in DCM. Deprotection of amine group of dehydrodipeptide esters containing dehydroalanine required the use of anisole (3 % v/v) for protection against oligomerization reactions. Mixture was stirred at room temperature for 30 min, and equivalent of *p*-toluenesulfonic acid was added. Mixing was continued for 15 min, and solvent was removed under reduced pressure. The residue was dissolved in dichloromethane, and solvent was carefully evaporated to remove the excess of trifluoroacetic acid. Products were crystallized from mixtures of isopropanol/hexane.

#### *Gly*-*ΔAla*-*OMe·Tos*

87 % yield (deprotection); mp = 151.5–155 °C with decomposition; ^1^H NMR δ 9.92 (s, 1H, N***H***), 8.02 (s, 3H, N***H***_3_^+^), 6.32 (s, 1H, C***H***_***A***_H_B ΔAla_), 5.84 (s, 1H, CH_A_***H***_***B***__ΔAla_), 3.80 (s, 2H, C***H***_2Gly_), 3.78 (s, 3H, OC***H***_3_). ^13^C NMR δ 166.14 (***C***=O_amid._), 163.45 (***C***=O_est._), 132.02 (***C***=), 110.58 (***C***H_2_=), 52.88 (O***C***H_3_), 41.10 (***C***H_2Gly_). HRMS (ESI) m/z calcd for C_6_H_11_N_2_O_3_ (M + H)^+^ 159.0764; found 159.0767; IR (KBr, cm^−1^) 3700–2600 broad (H-bonding), 1733 (C=O_ester_), 1689 IAB (C=O_amid_), 1634 (C=C), 1551 IIAB (C–N and N–H), 1200–1171 (C–O–C and SO_3_), 919 (=CH_2_).

#### *(S)Phe*-*ΔAla*-*OMe·Tos*

98 % yield (deprotection); mp = 156–157 °C with decomposition; ^1^H NMR δ 9.93 (s, 1H, N***H***), 8.25 (s, 3H, N***H***_3_^+^), 7.37–7.23 (m, 5H, Ar***H***_*P*he_), 6.27 (s, 1H, C***H***_***A***_H_B__ΔAla_), 5.85 (s, 1H, CH_A_***H***_***B***__ΔAla_), 4.42–4.34 (m, 1H, C***H***_Phe_), 3.76 (s, 3H, OC***H***_3_), 3.09 (ABX system, *J* 13.9, 6.1 Hz, 1H, C***H***_***A***_H_B Phe_), 2.99 (ABX system, *J* 13.9, 7.8 Hz, 1H, CH_A_***H***_***B***__Phe_). ^13^C NMR δ 168.13 (***C***=O_amid._), 163.37 (***C***=O_est._), 134.60 (***C***Ar_Phe_), 131.92 (***C***=), 129.58, 128.60, 127.32 (3 × ***C***Ar_Phe_), 111.52 (***C***H_2_=), 53.72 (***C***H_Phe_), 52.86 (O***C***H_3_), 37.10 (***C***H_2Phe_). HRMS (ESI) m/z calcd for C_13_H_17_N_2_O_3_ (M + H)^+^ 249.1234; found 249.1223; IR (KBr, cm^−1^) 3700–2700 broad (H-bonding), 1728 (C=O_ester_), 1694 IAB (C=O_amid_), 1638 (C=C), 1538 IIAB (C–N and N–H), 1203–1166 (C–O–C and SO_3_), 919 (=CH_2_).

#### *(S)Phe*-*ΔAla*-*OEt·Tos*

85 % yield (deprotection); mp = 139–141 °C; ^1^H NMR δ 9.91 (s, 1H, N***H***), 8.24 (s, 3H, N***H***_3_^+^), 7.37–7.24 (m, 5H, Ar***H***_Phe_), 6.27 (s, 1H, C***H***_***A***_H_B__ΔAla_), 5.84 (s, 1H, CH_A_H_B__ΔAla_), 4.44–4.35 (m, 1H, C***H***_Phe_), 4.22 (q, *J* = 7.1 Hz, 2H, OC***H***_**2**_CH_3_), 3.10 (ABX system, *J* = 13.9, 6.2 Hz, 1H, C***H***_***A***_H_B Phe_), 2.99 (ABX system, *J* = 13.9, 7.8 Hz, 1H, CH_A_***H***_***B***__Phe_), 1.25 (t, *J* = 7.1 Hz, 3H, OCH_2_C***H***_**3**_). ^13^C NMR δ 168.10 (***C***=O_amid._), 162.89 (***C***=O_est._), 134.60 (***C***Ar_Phe_), 132.10 (***C***=), 129.55, 128.60, 127.31 (3 × ***C***Ar_Phe_), 111.19 (***C***H_2_ =), 61.74 (***C***H_2_CH_3_), 53.70 (***C***H_Phe_), 37.10 (***C***H_2Phe_), 13.99 (CH_2_***C***H_3_). HRMS (ESI) m/z calcd for C_14_H_19_N_2_O_3_ (M + H)^+^ 263.1390; found 263.1395; IR (KBr, cm^−1^) 3700–2450 broad (H-bonding), 1713 (C=O_ester_), 1691 IAB (C=O_amid_), 1640 (C=C), 1535 IIAB (C–N and N–H), 1249–1167 (C–O–C and SO_3_), 915 (=CH_2_).

#### *(S)Phe*-*ΔAla*-*OPr*^i^*·Tos*

 80 % yield (deprotection); mp = 153–155 °C with decomposition; ^1^H NMR δ 9.88 (s, 1H, N***H***), 8.24 (s, 3H, N***H***_3_^+^), 7.39–7.21 (m, 5H, Ar***H***_Phe_), 6.25 (s, 1H, C***H***_***A***_H_B__ΔAla_), 5.81 (s, 1H, CH_A_***H***_***B***__ΔAla_), 5.00 (hept, J = 6.2 Hz, 1H, C***H***(CH_3_)_2_), 4.43–4.34 (m, 1H, C***H***_Phe_), 3.10 (dd, J = 13.9, 6.1 Hz, 1H, ABX system C***H***_***A***_H_B Phe_), 2.99 (dd, J = 13.9, 7.8 Hz, 1H, ABX system CH_A_***H***_***B*** Phe_), 1.26 (d, J = 6.2 Hz, 6H, CH(C***H***_3_)_2_). ^13^C NMR δ 168.07 (***C*** = O_amid._), 162.44 (***C*** = O_est._), 134.63 (***C***Ar_Phe_), 132.34 (***C***=), 129.55, 128.60, 127.31 (3 × ***C***Ar_Phe_), 110.91 (***C***H_2_=), 69.51 (***C***H(CH_3_)_2_), 53.70 (***C***H_Phe_), 37.11 (***C***H_2Phe_), 21.43 (CH(***C***H_3_)_2_). HRMS (ESI) m/z calcd for C_15_H_21_N_2_O_3_ (M + H)^+^ 277.1547; found 277.1545; IR (KBr, cm^−1^) 3700–2450 broad (H-bonding), 1710 (C=O_ester_), 1690 IAB (C=O_amid_), 1640 (C=C), 1534 IIAB (C–N and N–H), 1226–1169 (C–O–C and SO_3_), 919 (=CH_2_).

### Synthesis of allyl and propargyl esters of dipeptides containing dehydroalanine

A Cs_2_CO_3_ 0.163 g (0.5 mmol) was added to solution of Boc-Gly-ΔAla 0.244 g (1 mmol) or Boc-(S)Phe-ΔAla 0.334 g (1 mmol) in 5 mL of methanol. The mixture was stirred at room temperature for 2 h, and solvent was removed under reduced pressure. Solid residue was dissolved in 5 mL of THF for Boc-Gly-ΔAla or 5 mL of DMF for Boc-(S)Phe-ΔAla, and allyl bromide 0.856 mL (10 mmol) or propargyl bromide 1.114 mL (10 mmol) was added dropwise over 15 min. When peptide substrate was consumed (controlled by TLC), the solvent and excess of bromide were removed under reduced pressure. The residue was dissolved in 80 mL of ethyl acetate, filtrated and washed with: 1 M HCl (4 × 5 mL), saturated KHCO_3_ (4 × 5 mL) and brine. Organic layer was dried over MgSO_4_ and filtered, and 0.2 mL of anisole was added. The solvent was removed under reduced pressure at 35 °C. The residue was dissolved in 10 mL DCM, 1.5 mL of TFA was added and the mixture was stirred for 1 h at room temperature followed by addition of 0.190 g (1 mmol) of *p*-toluenesulfonic acid. Stirring was continued for additional 20 min, and solvent was removed under reduced pressure. The residue was evaporated two times with 20 mL of DCM to remove TFA excess. Products were crystallized from mixtures of isopropanol/hexane

#### *Gly*-*ΔAla*-*OAll·Tos*

72 % global yield; mp = 159–161.5 °C with decomposition; ^1^H NMR δ 9.92 (s, 1H, N***H***), 8.04 (s, 3H, N***H***_3_^+^), 6.34 (s, 1H, C***H***_***A***_H_B__ΔAla_), 6.05–5.92 (m, 1H, CH_2_=C***H***), 5.88 (s, 1H, CH_A_***H***_***B***__ΔAla_), 5.40–5.33 (2 × m, 1H, CH=C***H***_***A***_H_B_), 5.30–5.25 (2 × m, 1H, CH=CH_A_**H**_**B**_), 4.73 (m, 2H, OC***H***_2_), 3.81 (s, 2H, C***H***_2Gly_). ^13^C NMR δ 166.11 (***C***=O_amid._), 162.63 (***C***=O_est._), 132.09 (***C***H=_All_), 132.01 (***C***=), 118.37 (***C***H_2_=_All_), 110.75 (***C***H_2_=_ΔAla_), 65.92 (O***C***H_2_), 41.08 (***C***H_2Gly_). HRMS (ESI) m/z calcd for C_8_H_13_N_2_O_3_ (M + H)^+^ 185.0921; found 185.0919. IR (KBr, cm^−1^) 3600–2600 broad (H-bonding), 1718 (C=O_ester_), 1692 IAB (C=O_amid_), 1649 (C=C), 1538 IIAB (C–N and N–H), 1198 broad (C–O–C and SO_3_), 922 (=CH_2_).

#### *Gly*-*ΔAla*-*OPrg·Tos*

71 % global yield; mp = 141–143.5 °C with decomposition; ^1^H NMR δ 9.98 (s, 1H, N***H***), 8.05 (s, 3H, N***H***_3_^+^), 6.36 (s, 1H, C***H***_***A***_H_B__ΔAla_), 5.87 (s, 1H, CH_A_***H***_***B***__ΔAla_), 4.89 (d, *J* = 2.3 Hz, 2H, OC***H***_2_), 3.81 (s, 2H, C***H***_2Gly_), 3.67 (t, *J* = 2.3 Hz, 1H, ≡C***H***). ^13^C NMR δ 166.20 (***C***=O_amid._), 162.30 (***C***=O_est._), 131.69 (***C***=), 111.49 (***C***H_2_=), 78.48, 77.92 (2 × ***C***≡***C***H), 53.34 (O***C***H_2_), 41.10 (***C***H_2Gly_). HRMS (ESI) m/z calcd for C_8_H_11_N_2_O_3_ (M + H)^+^ 183.0764; found 183.0771. IR (KBr, cm^−1^) 3600–2800 broad (H-bonding), 2129 (C≡C), 1732 (C=O_ester_), 1700 IAB (C=O_amid_), 1638 (C=C), 1547 IIAB (C–N and N–H), 1178 broad (C–O–C and SO_3_), 895 (=CH_2_).

#### *(S)Phe*-*ΔAla*-*OAll·Tos*

70 % global yield; mp = 123.5–125 °C with decomposition; ^1^H NMR δ 9.96 (s, 1H, N**H**), 8.24 (s, 3H, N***H***_3_^+^), 7.39–7.23 (m, 5H, Ar***H***_Phe_), 6.30 (s, 1H, C***H***_***A***_H_B__ΔAla_), 6.03–5.91 (m, 1H, CH_2_=C***H***), 5.89 (s, 1H, CH_A_***H***_***B***__ΔAla_), 5.40–5.33 (2 × m, 1H, CH=C***H***_*A*_H_B_), 5.30–5.25 (2 × m, 1H, CH=CH_A_***H***_***B***_), 4.71 (m, 2H, OC***H***_2_), 4.40 (wide s, 1H, C***H***_Phe_), 3.10 (dd, J = 13.9, 6.2 Hz, 1H, ABX system C***H***_***A***_H_B Phe_), 3.00 (dd, J = 13.9, 7.8 Hz, 1H, ABX system CH_A_***H***_***B***__Phe_). ^13^C NMR δ 168.15 (***C***=O_amid._), 162.57 (***C***=O_est._), 134.59 (***C***Ar_Phe_), 132.08 (***C***H=_All_), 131.90 (***C***=), 129.55, 128.59, 127.31 (3 × ***C***Ar_Phe_), 118.39 (***C***H_2_=_All_), 111.71 (***C***H_2_=_ΔAla_), 65.92 (O***C***H_2_), 53.70 (***C***H_Phe_), 37.09 (***C***H_2Phe_). HRMS (ESI) m/z calcd for C_15_H_19_N_2_O_3_ (M + H)^+^ 275.1390; found 275.1381. IR (KBr, cm^−1^) 3600–2700 broad (H-bonding), 1722 (C=O_ester_), 1699 IAB (C=O_amid)_, 1637 (C=C), 1527 IIAB (C–N and N–H), 1231–1176 (C–O–C and SO_3_), 947 (=CH_2_).

#### *(S)Phe*-*ΔAla*-*OPrg·Tos*

65 % global yield; mp = 170–172 °C with decomposition; ^1^H NMR δ 10.02 (s, 1H N***H***), 8.24 (s, 3H, N***H***_3_^+^), 7.39–7.24 (m, 5H, Ar***H***_Phe_), 6.30 (s, 1H, C***H***_***A***_H_B__ΔAla_), 5.89 (s, 1H, CH_A_***H***_***B***__ΔAla_), 4.87 (d, J = 2.3 Hz, 2H, OC***H***_2_), 4.38 (wide s, 1H, C***H***_Phe_), 3.68 (t, J = 2.3 Hz, 1H, ≡ C***H***), 3.11 (dd, J = 13.9, 6.0 Hz, 1H, ABX system C***H***_**A**_H_B Phe_), 3.00 (dd, J = 13.9, 7.8 Hz, 1H, ABX system CH_A_***H***_***B***__Phe_). ^13^C NMR δ 168.20 (***C***=O_amid._), 162.22 (***C***=O_est._), 134.58 (***C***Ar_Phe_), 131.58 (***C***=), 129.56, 128.61, 127.34 (3 × ***C***Ar_Phe_), 112.52 (***C***H_2_=_ΔAla_), 78.48, 77.89 (2 × ***C*** ≡ ***C***H), 53.72 (***C***H_Phe_), 53.32 (O***C***H_2_), 37.07 (***C***H_2Phe_). HRMS (ESI) m/z calcd for C_15_H_17_N_2_O_3_ (M + H)^+^ 273.1234; found 273.1224. IR (KBr, cm^−1^) 3600–2850 broad (H-bonding), 2120 (C≡C), 1745 (C=O_ester_), 1699 IAB (C=O_amid_), 1632 (C=C), 1517 IIAB (C–N and N–H), 1227–1168 broad (C–O–C and SO_3_).

### Synthesis of allyl and propargyl esters of dipeptides containing (Z)-dehydrophenylalanine

Boc-Gly-Δ^Z^Phe 0.320 g (1.0 mmol) was dissolved in 5 mL DMF, and Cs_2_CO_3_ 0.163 g (0.5 mmol) was added. Mixture was stirred for 3 h, and allyl bromide 0.856 mL (10 mmol) or propargyl bromide 1.114 mL (10 mmol) was added dropwise over 15 min. The reaction was continued for 12 h stirring at room temperature. Further steps of synthesis were done according to procedure described for allyl and propargyl esters of Boc-Gly-ΔAla. The deprotection reaction of amine group was performed without addition of anisole and *p*-toluenesulfonic acid.

#### *Gly*-*Δ*^*Z*^*Phe*-*OAll·TFA*

88 % global yield; mp = 137–138.5 °C with decomposition; ^1^H NMR δ 10.19 (s, 1H, N***H***), 8.19 (s, 3H, N***H***_3_^+^), 7.78–7.41 (m, 5H, Ar***H***_ΔPhe_), 7.39 (s, 1H, C**H**_ΔPhe_), 6.08–5.90 (m, 1H, CH_2_=C***H***), 5.43–5.34 (2 × m, 1H, CH=C***H***_***A***_H_B_), 5.30–5.23 (2 × m, 1H, H=CH_A_***H***_***B***_), 4.69 (m, 2H, OC***H***_2_), 3.81 (s, 2H, C***H***_2Gly_). ^13^C NMR δ 166.22 (***C***=O_amid._), 164.08 (***C***=O_est._), 133.11, 132.87, 132.44, 130.18, 129.89, 128.79, 124.87, 117.99 (8 C atoms derived from (Z)-dehydrophenylalanine and allyl group), 65.54 (O***C***H_2_), 40.38 (***C***H_2Gly_), (peaks derived from TFA group are omitted for clarity). HRMS (ESI) m/z calcd for C_14_H_17_N_2_O_3_ (M + H)^+^ 261.1234; found 261.1229. IR (KBr, cm^−1^) 3600–2600 broad (H-bonding), 1723 (C=O_ester_), 1698 IAB (C=O_amid_), 1625 (C=C), 1529 IIAB (C–N and N–H), 1201–1180 (C–O–C), 922 (=CH_2_), 837 (=CH_ΔPhe_).

#### *Gly*-*Δ*^*Z*^*Phe*-*OPrg·TFA*

92 % global yield; mp = 145–147 °C with decomposition; ^1^H NMR δ 10.21 (s, 1H, N***H***), 8.20 (s, 3H, N***H***_3_^+^), 7.73–7.42 (m, 5H, Ar***H***_ΔPhe_), 7.40 (s, 1H, C***H***_ΔPhe_), 4.84 (d, *J* = 2.4 Hz, 2H, OC***H***_2_), 3.81 (s, 2H, C***H***_2Gly_), 3.64 (t, *J* = 2.4 Hz, 1H, ≡ C***H***). ^13^C NMR δ 166.24 (***C***=O_amid._), 163.72 (***C***=O_est._), 133.88, 132.74, 130.27, 130.07, 128.83, 124.33 (6 C atoms derived from (Z)-dehydrophenylalanine), 78.29, 78.16 (2 × ***C***≡***C***H), 52.86 (O***C***H_2_), 40.38 (***C***H_2Gly_) (for clarity peaks derived from TFA group are omitted); HRMS (ESI) m/z calcd for C_14_H_15_N_2_O_3_ (M + H)^+^ 259.1077; found 259.1060. IR (KBr, cm^−1^) 3600–2600 broad (H-bonding), 2132 (C≡C), 1723 (C=O_ester_), 1698 IAB (C=O_amid_), 1624 (C=C) 1531 IIAB (C–N and N–H), 1201–1179 (C–O–C), 837 (=CH_ΔPhe_).

### Efforts to synthesize dehydrodipeptide glycidyl esters

*Method I* Boc-Gly-Δ^Z^Phe 0.160 g (0.5 mmol), Et_3_N 0.196 mL (1.1 mmol) and (*S*)-glycidol 0.266 mL (2.0 mmol) were dissolved in 2.0 mL of acetonitrile, and TBTU (Abdelmoty *et al.*, 1994) 0.208 g (0.65 mmol) was then added. Mixture was stirred at room temperature for 2.5 h, and solvent removed under reduced pressure. The residue was dissolved in 70 mL of ethyl acetate and washed subsequently with: 1 M HCl (3 × 5 mL), saturated KHCO_3_ (3 × 5 mL) and brine. Organic phase was dried over MgSO_4_ and filtered, and solvents were removed. We were unable to purify a mixture of products obtained using column chromatography with silica gel 60H (Merck) as stationary phase and various eluents. Thus, crude mixture was used in deprotection step. HRMS (ESI) indicated the presence of the desired product as a major one: m/z calcd for C_19_H_24_N_2_O_6_ (M + Na)^+^ 399.1526; found 399.1529.

*Method II iso*-butyl chloroformate 0.066 mL (0.5 mmol) was added to solution of Boc-Gly-Δ^Z^Phe 0.160 g (0.5 mmol) and Et_3_N 0.070 mL (0.5 mmol) in dichloromethane when cooling in ice bath to −15 °C. After 1.5 min, glycidol 0.133 mL (1.0 mmol) was added. The mixture was left to warm to room temperature, and stirring was continued for next 24 h. Further steps of synthesis were performed according to the methodology described for Method I and afforded similar mixture of products.

#### Deprotection of amine group

*Method I* Trifluoroacetic acid 0.5 mL was added to solution of Boc-Gly-Δ^Z^Phe-OGdl 0.098 g (0.25 mmol) in 2 mL of dichloromethane. Mixture was stirred for 20 min at room temperature, and solvent was removed under reduced pressure. The residue was evaporated three times with 20 mL of dichloromethane and 20 mL of diethyl ether to remove the excess of trifluoroacetic acid. Mixture of products was obtained as oily residue. HRMS (ESI) indicated the presence of the two major products—desired glycidol ester (Gly-Δ^Z^Phe-OGdl(S)) and the product of oxirane ring opening—Gly-Δ^Z^Phe-OCH_2_CH(OH)CH_2_OH: m/z calcd for C_14_H_17_N_2_O_4_ (M + H)^+^ 277.1183 and C_14_H_19_N_2_O_5_ (M + H)^+^ 295.1288; found 277.1164 and 295.1266, respectively.

*Method II* HCl in methanol (~3.8 M) solution was prepared by bubbling dry HCl gas through methanol for 1 h at 0 °C. Crude Boc-Gly-Δ^Z^Phe-OGdl(S) 0.129 g (0.34 mmol) was dissolved in methanol (1.2 mL), and HCl–methanol solution was added (1.3 mL). After 1 h at room temperature, solvent was evaporated under reduced pressure. The oil residue was evaporated three times with 5 mL of dichloromethane. Product was crystallized from mixture of isopropanol/diethyl ether/hexane (2:1), filtered and dried *in vacuo*.

In that manner, *Gly*-*Δ*^*Z*^*Phe*-*OCH*_*2*_*CH(OH)CH*_*2*_*Cl·HCl* was obtained as a white solid in 50 % yield (deprotection): mp = 178–180 °C decomposition; ^1^H NMR δ 10.28 (s, 1H, N***H***), 8.30 (s, 3H, N***H***_3_^+^), 7.74–7.40 (2 × m, 2H and 4H, Ar***H***_Δ(Z)Phe_ overlapped with C***H***_Δ(Z)Phe_), 5.67 (d, *J* = 5.1 Hz, 1H, O***H***), 4.18 (dd, *J* = 11.1, 5.2 Hz, 1H), 4.13 (dd, *J* = 11.1, 5.7 Hz, 1H), 4.05–3.97 (m, 1H, C***H***OH), 3.79 (s, 2H, CH_2Gly_), 3.73 (dd, *J* = 11.3, 4.8 Hz, 1H), 3.66 (dd, *J* = 11.3, 5.5 Hz, 1H). Four dd at 4.18, 4.13, 3.73, 3.66 ppm derived from two CH_2_ groups which are present at OC***H***_**2**_CH(OH)C***H***_**2**_Cl part of the molecule. ^13^C NMR δ 166.21 (***C***=O_amid._), 164.22 (***C***=O_est._), 133.44, 132.91, 130.24, 129.87, 128.78, 124.62 (6 C atoms derived from (Z)-dehydrophenylalanine), 67.94, 66.14, 46.56 (O***C***H_2_***C***H(OH)***C***H_2_Cl), 40.37 (***C***H_2Gly_). HRMS (ESI) m/z calcd for C_14_H_18_ClN_2_O_4_ (M + H)^+^ 313.0950; found 313.0950; intensity of ions: 313.0950 I = 100 %; 315.0926 I = 34.2 % (chlorine isotopes). IR (KBr, cm^−1^) 3600–2550 broad (H-bonding), 1706 (C=O_ester_), 1680 IAB (C=O_amid_), 1636 (C=C), 1541 IIAB (C–N and N–H), 841 (=CH_ΔPhe_).

### Enzymatic studies

Cathepsin C was isolated from bovine spleen by modified method of McDonald *et al.* (1972). The K_M_ value of 2.3 mM for the enzyme was measured using synthetic substrate—glycine-*L*-phenylalanine-*p*-nitroanilide (Gly-*L*-Phe-*p*NA). Purity of the enzyme was confirmed by electrophoresis.

### Inhibitory studies

Cathepsin C was activated for 0.5 h in a water bath at 37 °C in 1 % NaCl solution containing 1 mM EDTA-Na_2_ and 5 mM 2-mercaptoethanol. The enzymatic reaction was carried out at 37 °C in 100 mM acetate buffer, pH 5.0, containing 1 mM EDTA-Na_2_, 1 mM DTT and 30 mM NaCl (all final concentrations). The progress of the reaction was monitored spectrophotometrically (UV–Vis spectrophotometer Cintra 303) at a wavelength of 405 nm against a control sample containing no enzyme. Attempting mixture contained: solution of the synthetic substrate Gly-*L*-Phe-*p*NA in acetate buffer at pH 5 containing 1 mM EDTA-Na_2_, 1 mM DTT, 30 mM NaCl (substrate concentration: 2.7–0.01 mM—final concentration), the solution of inhibitor in reaction buffer (concentration of compound depended on inhibitory potential), and enzyme.

Kinetic constants K_M_, V_max_ and K_i_ and type of inhibition were determined by using Lineweaver–Burk, Dixon, Hanes-Woolf and half-inhibitory concentration methods using the computer program provided kindly by dr Józef Hurek (University of Opole). The *K*_i_ values presented in the Table 1 are the average ones calculated by using all these methods. All measurements were taken in a three repetitions.

**Table 1:** Inhibitory constants of the studied dehydrodipeptides toward cathepsin C

Compound	*K*_i_ (μM)	Compound	K_i_ (μM)
(*S*)Phe-AlaOMe*·*Tos	416 ± 10		
Gly-ΔAlaOMe*·*Tos	NI	(*S*)Phe-ΔAlaOMe*·*Tos	64 ± 3
(*S*)Phe-ΔAlaOEt*·*Tos	84 ± 4	(S)Phe-ΔAlaOPr^*i*^ *·*Tos	171 ± 8
Gly-ΔAlaOAll*·*Tos	460 ± 20	Gly-^Z^ΔPheOAll*·*TFA	13 ± 1
(*S*)Phe-ΔAlaOAll*·*Tos	17 ± 1		
Gly-ΔAlaOPrg*·*Tos	320 ± 20	Gly-^Z^ΔPheOPrg*·*TFA	33 ± 2
(*S*)Phe-ΔAlaOPrg*·*Tos	86 ± 4	Gly-Δ^Z^PheO-CH_2_CH(OH)CH_2_Cl*·*HCl	5.5 ± 0.5

NI—no inhibition up to 1245 mM

### Molecular modeling

The structures of studied dehydropeptides were optimized in Gaussian09 program at the B3LYP/6-311 g (d,p) level (Frisch *et al.*, 2004) in gas phase with using Merz-Singh-Kollman scheme (Besler *et al.*, 1990) to the determination of the atomic charges. The calculations of the docking process were performed using AutoDock program (Morris *et al.*, 2009). The starting geometry and charges of the dehydropeptides were taken from the ab initio calculations. The structure of cathepsin C was extracted from the structure of human dipeptidyl peptidase I deposited EC 3.4.14 in Protein Data Bank (Turk *et al.*, 2001). Structure of the enzyme has been protonated on the H++ server (Myers *et al.*, 2006) at pH = 5.7, and also charges of all enzymatic atoms have been assignment on this server. During the docking process, main chain of the dehydropeptide was fixed, whereas side chains and the terminal groups were left as flexible. The coordinates of the SH proton from the Cys234 were taken as a grid center in the docking process. In the simulation, docking process was performed 100 times. Analysis of the obtained results has been performed by using AutoDock Tools (Morris *et al.*, 2009).

## Results and discussion

Cathepsin C (EC 3.4.14.1) is a lysosomal cysteine protease expressed in majority of mammalian tissues and is primarily responsible for activation of serine proteases in inflammatory and immune cells (Reiser *et al.*, 2010). It sequentially removes dipeptides from the *N*-termini of protein and peptide substrates (Lindley, 1972; Poręba *et al.*, 2014). Increasing evidence of the key role of DPPI in various diseases, such as sepsis, asthma, Duchenne muscular dystrophy, rheumatoid arthritis, basal cell carcinomas, chronic obstructive pulmonary disease and other inflammatory disorders (Guay *et al.*, 2010; Laine and Busch-Petersen, 2010), stimulates interest in this enzyme as the possible medicinal target.

Dehydropeptides appear to be weak inhibitors of the enzyme (Latajka *et al.*, 2006, 2008). In this paper, we synthesized series of structurally variable esters of glycyl^Z^dehydrophenylalanine and its analogs. We speculated that the possible binding of the aromatic part of the inhibitor within S2 pocket of the enzyme might result in reaction between active ester (allyl, propargyl or glycidyl) with thiol moiety of the active-site cysteine. Unfortunately, obtained compounds exerted moderate inhibitory activity acting as competitive inhibitors. More likely this results from different than expected binding mode of these compounds.

### Synthesis of inhibitors

**Fig. 1:**
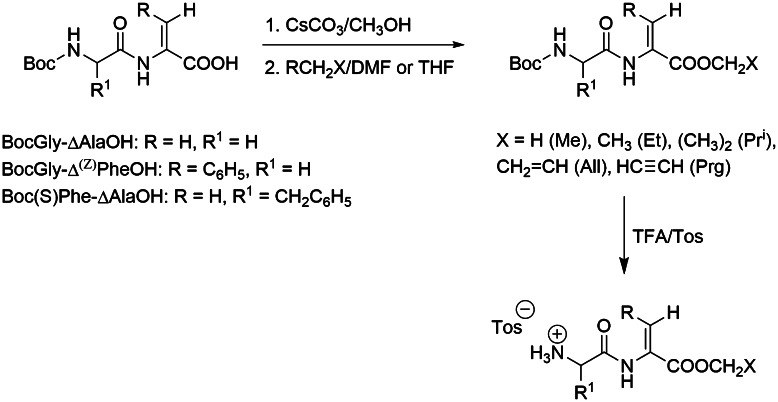
Synthesis of dehydrodipeptide methyl, ethyl, isopropyl, allyl and propargyl esters

**Fig. 2:**
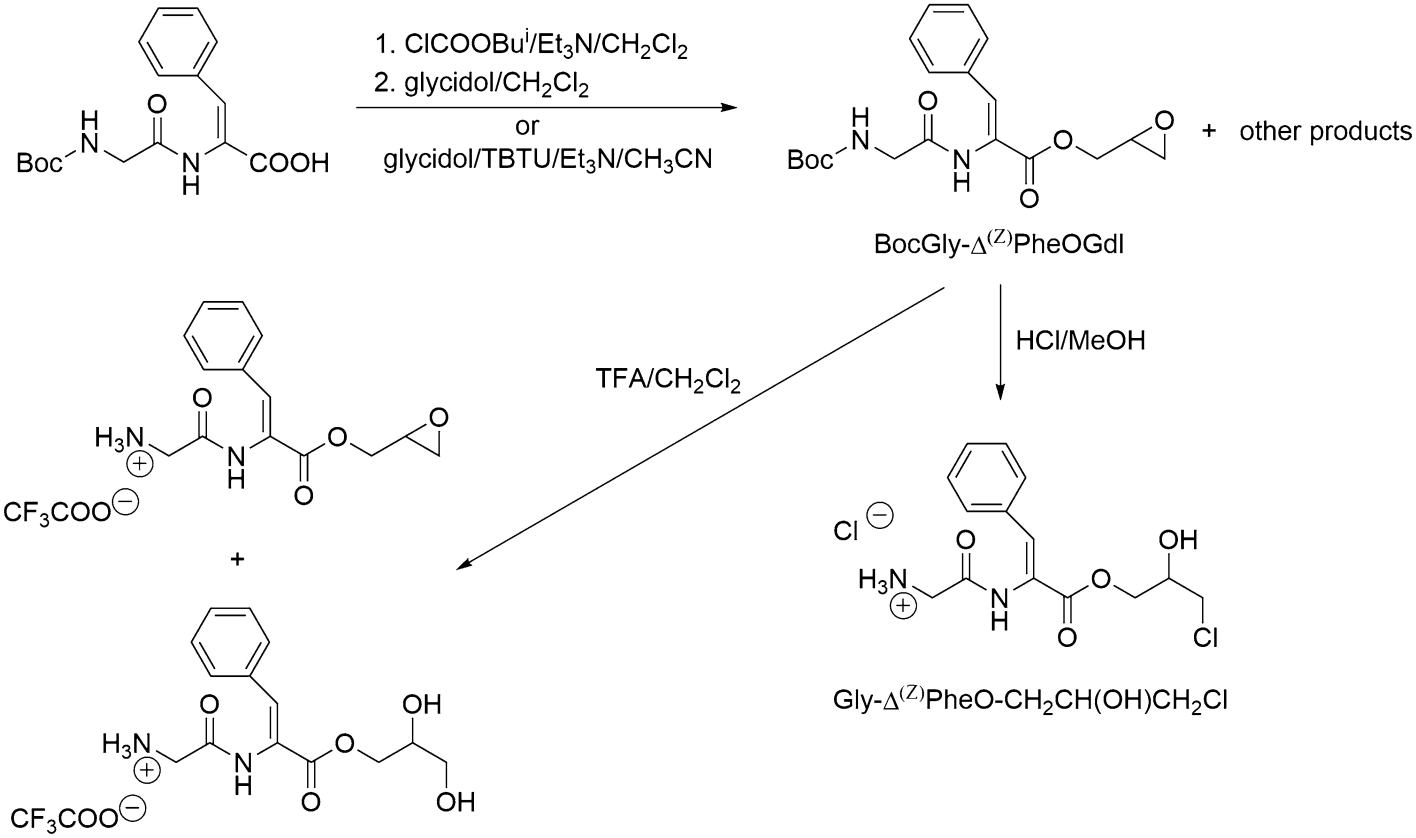
Synthesis of dehydrodipeptide glycidyl ester

Esters of dehydropeptides have been synthesized using classical methods of peptide chemistry. The synthetic schemes are outlined in Figs. 1 and 2. As seen from the figures for each group of esters, specific method of their preparation should be elaborated. Direct esterification of Boc-Gly-ΔAla with DMTMM (Kunishima *et al.*, 1999) as coupling agent gave non-satisfactory results (30 % of yield). Far better results for esterification of Boc-Gly-ΔAla were obtained via nucleophilic substitution of alkyl halides with dipeptide cesium salts (Fig. 1). This method gives product with yield 91 %. Glycidyl esters seem to be more interesting as inhibitors of cathepsin since they posses oxirane ring, which is known to react preferably with the enzyme active-site cysteine. In order to prepare these esters, two standard methods, both basing on the activation of carboxylic moiety, have been elaborated (Fig. 2). Unfortunately, the reaction afforded inseparable mixture of glycidyl ester and some products of oxirane ring opening. Efforts to remove Boc protection by trifluoroacetic acid were unsuccessful and gave even more complex mixture of products, whereas using hydrogen chloride in methanol we were able to isolate 3-chloro-2-hydroxypropyl ester of Boc-Gly-Δ^Z^Phe.

### Inhibitory studies

**Fig. 3:**
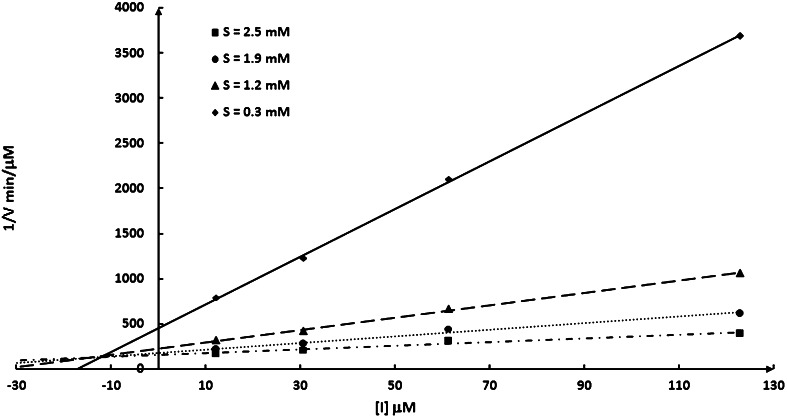
Dixon plot for the hydrolysis Gly-Phe-*p*-NA by bovine cathepsin C versus increasing concentration of Gly-^Z^ΔPheOAll

Inhibitory activities of the synthesized esters are collected in Table 1 and compared to action of methyl *L*-phenylalanyl-*L*-alaninate (Phe-AlaOMe). All the compounds appeared to be competitive inhibitors, as shown in Fig. 3 for Gly-^Z^ΔPheOAll trifluoroacetate as a representative example. The most active appeared to be Gly-Δ^Z^Phe-OCH_2_CH(OH)CH_2_Cl*·*HCl, Gly-^Z^ΔPheOAll*·*TFA and (*S*)Phe-ΔAlaOAll*·*Tos, which inhibitory constants were in micromolar range. Quite interesting, six of the peptides—Gly-^Z^ΔPheOPrg·TFA, Phe-ΔAlaOMe·Tos, Phe-ΔAlaOEt·Tos, (S)Phe-ΔAlaOPr^*i*^*·*Tos, (*S*)Phe-ΔAlaOAll*·*Tos and (*S*)Phe-ΔAlaOPrg*·*Tos—inhibit cathepsin C according to slow-binding mechanism. This mechanism is of B type and considers conformational rearrangement of inhibitor after binding to the enzyme (Pawełczak and Hurek, 2014). From the data shown in Table 1, it is also not possible to derive clear-cut structure–activity relationship. Contrary to recent studies on the structural requirements for the specific substrates for cathepsin C (Poręba *et al.*, 2014), introduction of *N*-terminal phenylalanine into peptide chain results in elevation of affinity of Phe-ΔAlaOMe·Tos if compared with Gly-ΔAlaOMe·Tos. This suggests that both dipeptide and dehydrodipeptide esters are bound differently than synthetic substrate of this enzyme.

Therefore, simple studies on their presumable binding using AutoDock program had been undertaken.

### Molecular modeling

**Fig. 4:**
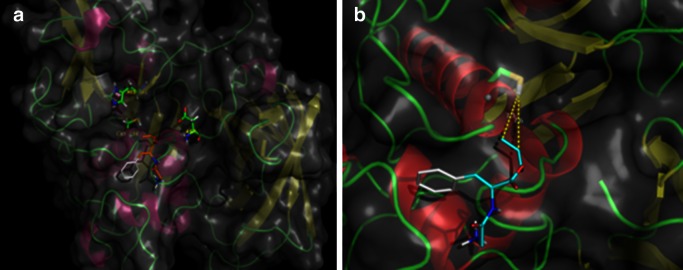
**a** Most probable binding mode of Gly-^Z^ΔPheOAll by cathepsin C and found by molecular modeling. Catalytic triad is shown in *green,* whereas inhibitor in *white* and *gold*. **b** Distance of allylic group of inhibitor from thiol moiety of active-site cysteine

Simple molecular modeling using AutoDock has shown that dehydrodipeptide esters are bound at the surface of the enzyme in a non-typical manner. Their phenyl rings are not, as expected, submerged in the cathepsin C cavity responsible for binding aromatic fragments of the substrates and inhibitors but are rather placed at the surface of the enzyme. The most probable binding mode of Gly-^Z^ΔPheOAll is shown in Fig. 4. As seen from this figure, allylic double bond of the inhibitor, albeit directed toward cathepsin C active-site cysteine 234, is too far away from thiol moiety (7–9 Å) to form a covalent adduct. This non-typical pattern of binding of dehydrodipeptide esters found from calculations well explains moderate inhibitory activity of these compounds.

## Conclusions

Synthesis of esters of dehydropeptides is not an easy task and requires the choice of specific method tailored to each case. Esters of dehydrodipeptides containing C-terminal dehydroalanine or (Z)-dehydrophenylalanine appeared to be moderate or weak inhibitors of cathepsin C. As suggested by molecular modeling, they are bound rather on the surface of the enzyme than inside of the binding cavities of the enzyme.

## Electronic supplementary material

Supplementary material 1 (DOC 1159 kb)
