# Supplementary material for: Synthesis of dehydrodipeptide esters and their evaluation as inhibitors of cathepsin C
Source: Med Chem Res. 2015 Apr 16;24(8):3157–65. doi: 10.1007/s00044-015-1366-0 (PMC4500854; doi:10.1007/s00044-015-1366-0)
Supplement: Supplementary file 1 — Supplementary material 1 (DOC 1159 kb) [file 44_2015_1366_MOESM1_ESM.doc]

**Supplementary material**

**Synthesis of dehydrodipeptide esters and their evaluation as inhibitors of cathepsin C**

**Maciej Makowski^1^,* Paweł Lenartowicz^1^, Bartosz Oszywa^1^, Michał Jewgiński^2^, Małgorzata Pawełczak^1^ and Paweł Kafarski ^1,2^**

*^1^Faculty of Chemistry, Opole University, Oleska 48, 45-052 Opole, Poland ^2^Faculty of Chemistry, Department of Bioorganic Chemistry, Wroclaw University of Technology, Wybrzeze Wyspianskiego 27, 50-370 Wroclaw, Poland*

**^1^H NMR spectrum of Gly-ΔAla-OMe·Tos**


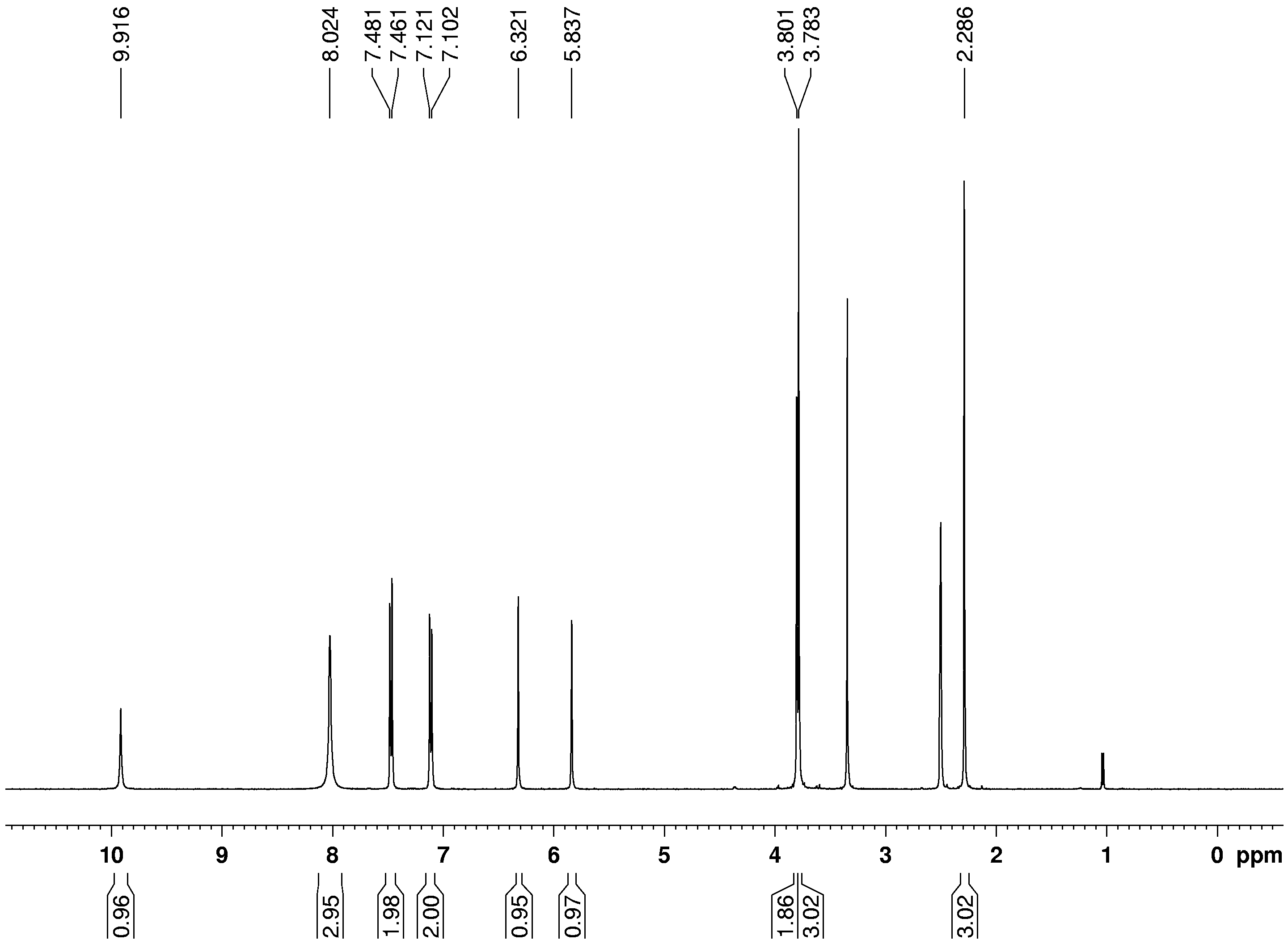


**^13^C NMR spectrum of Gly-ΔAla-OMe·Tos**


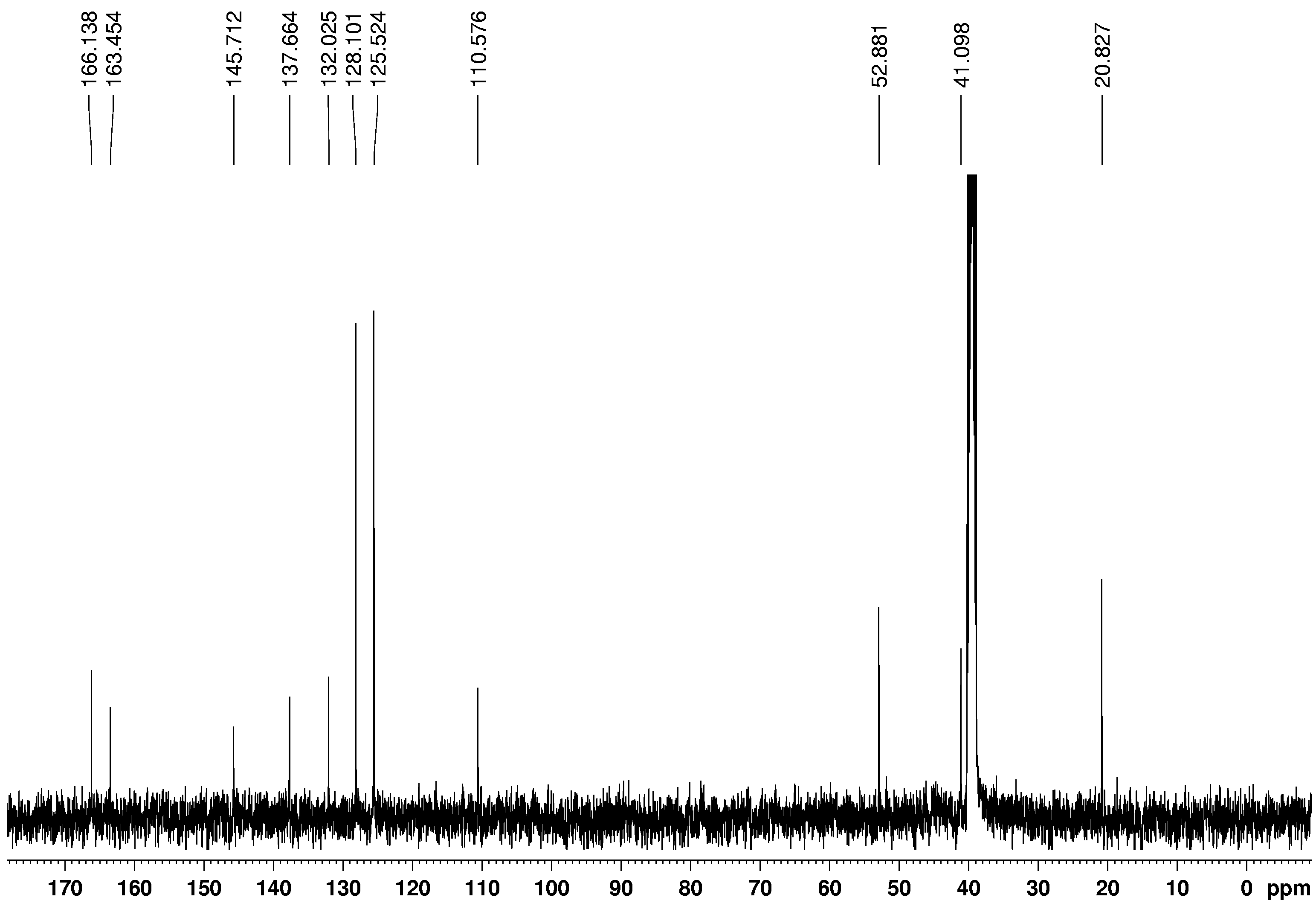


**^1^H NMR spectrum of (S)Phe-ΔAla-OMe·Tos** (methanol is seen as impurity)


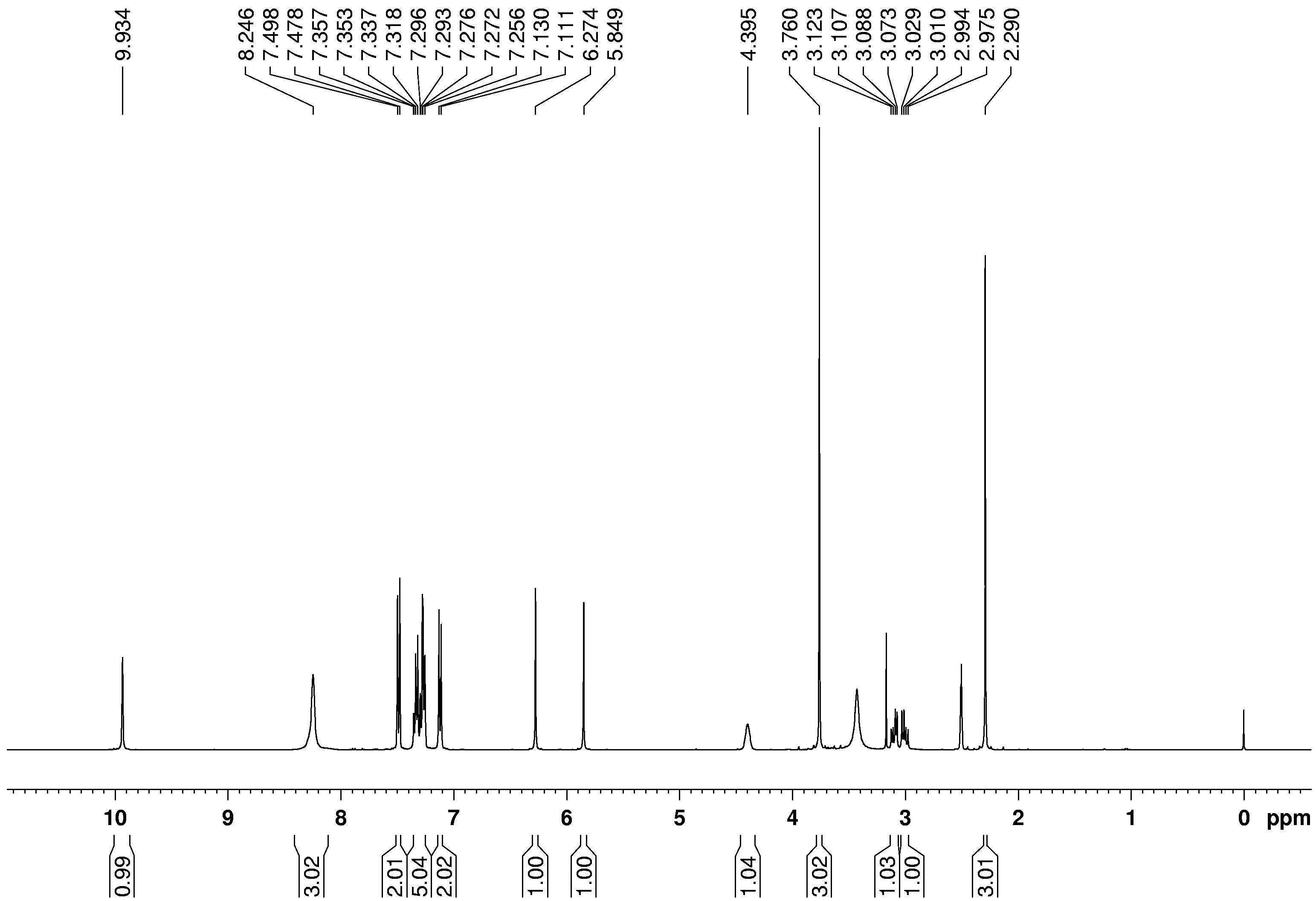


**^13^C NMR spectrum of(S)Phe-ΔAla-OMe·Tos** (methanol is seen as impurity)


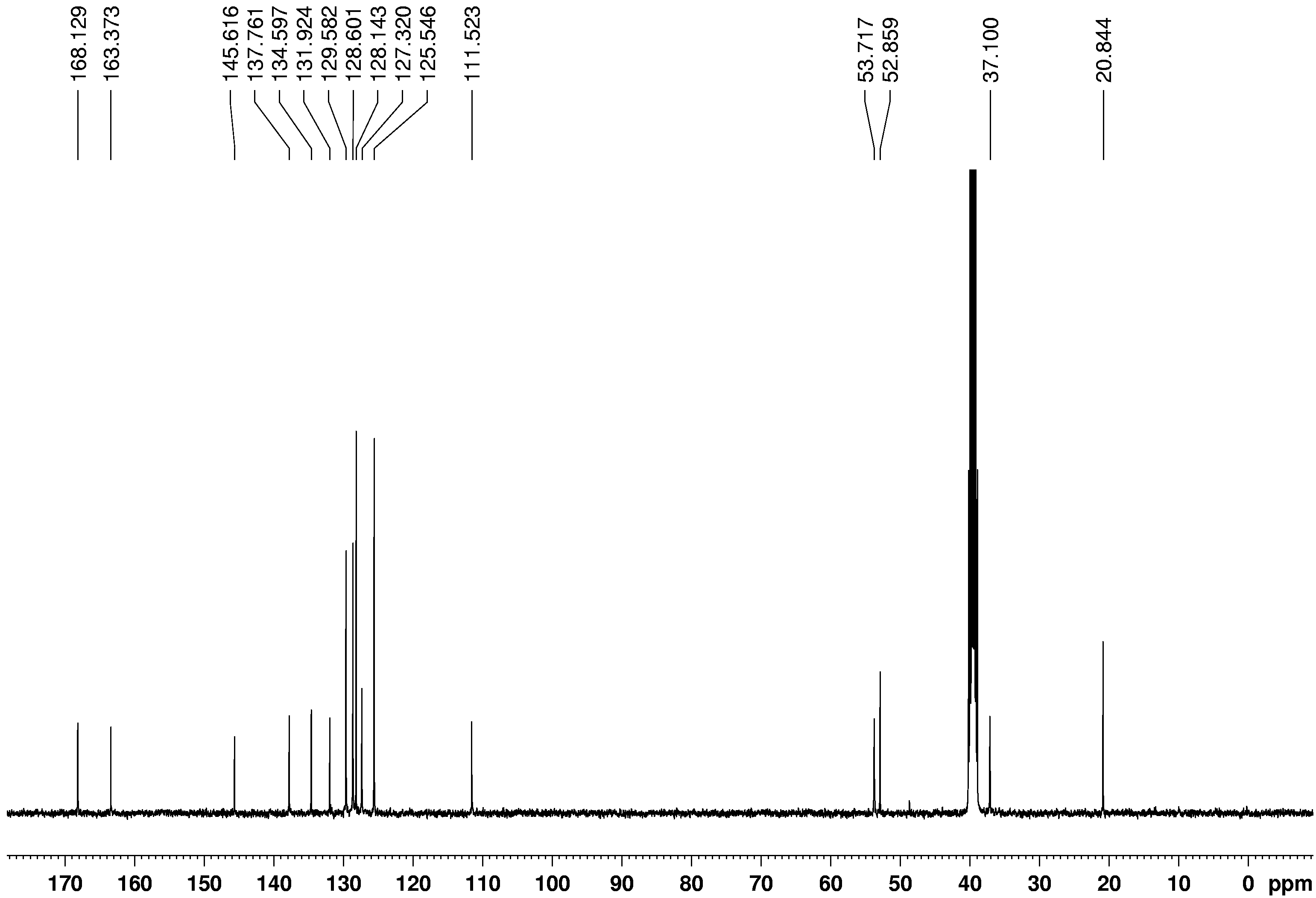


**^1^H-^13^C HSQC NMR spectrum of (S)Phe-ΔAla-OMe·Tos** (methanol is seen as impurity)


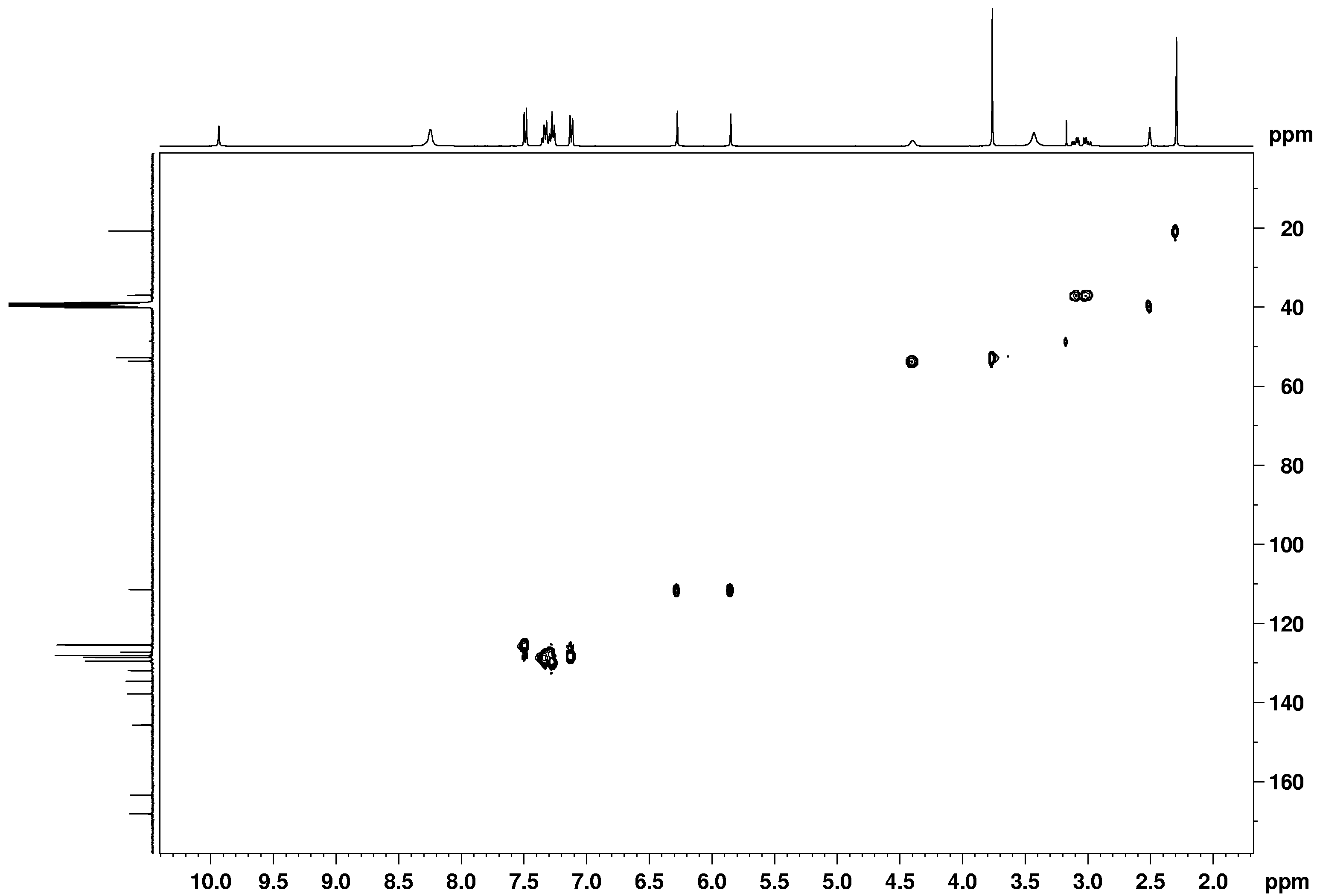


**^1^H-^13^C HMBC NMR spectrum of (S)Phe-ΔAla-OMe·Tos** (methanol is seen as impurity)


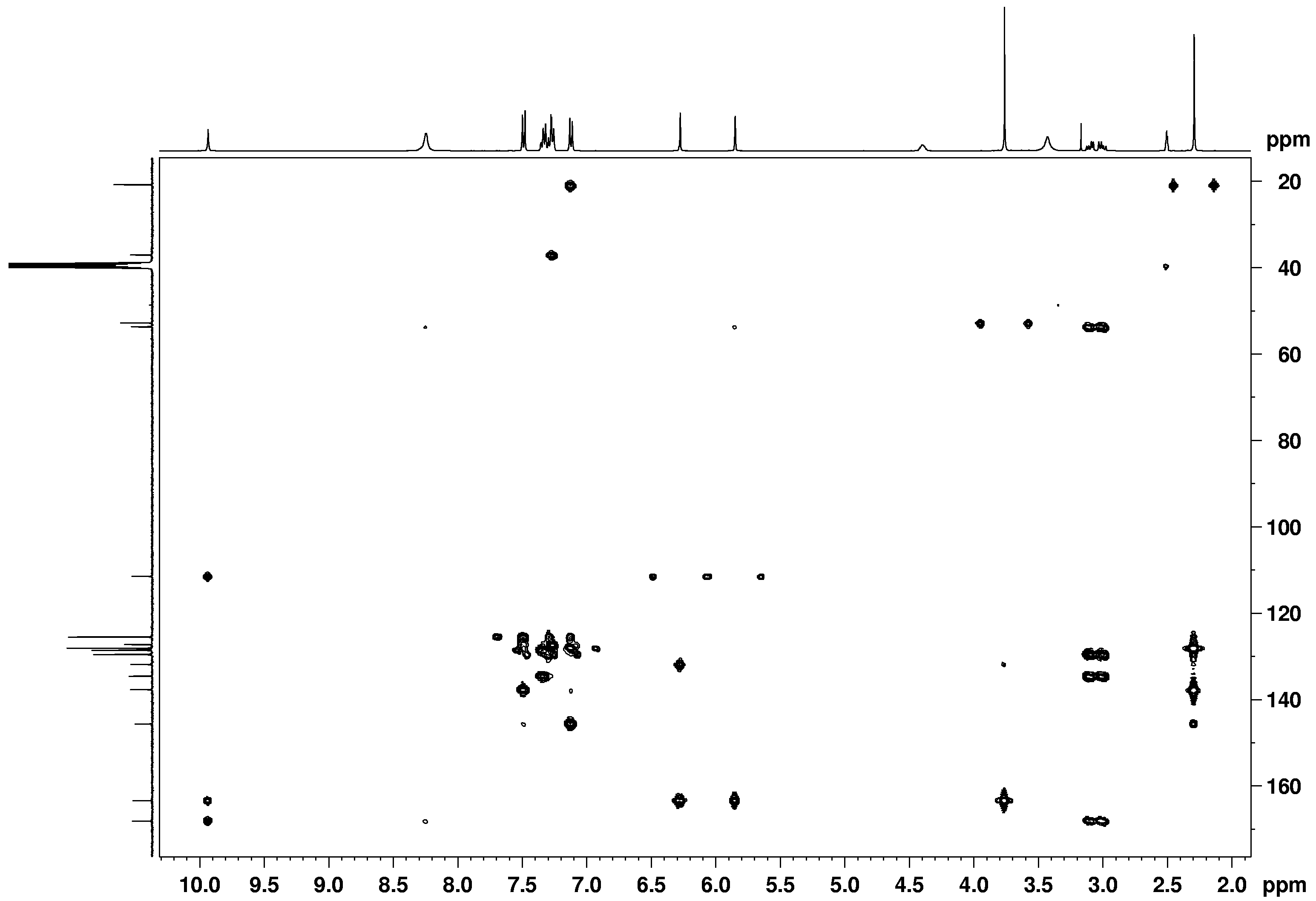


**^1^H NMR spectrum of (S)Phe-ΔAla-OEt·Tos**


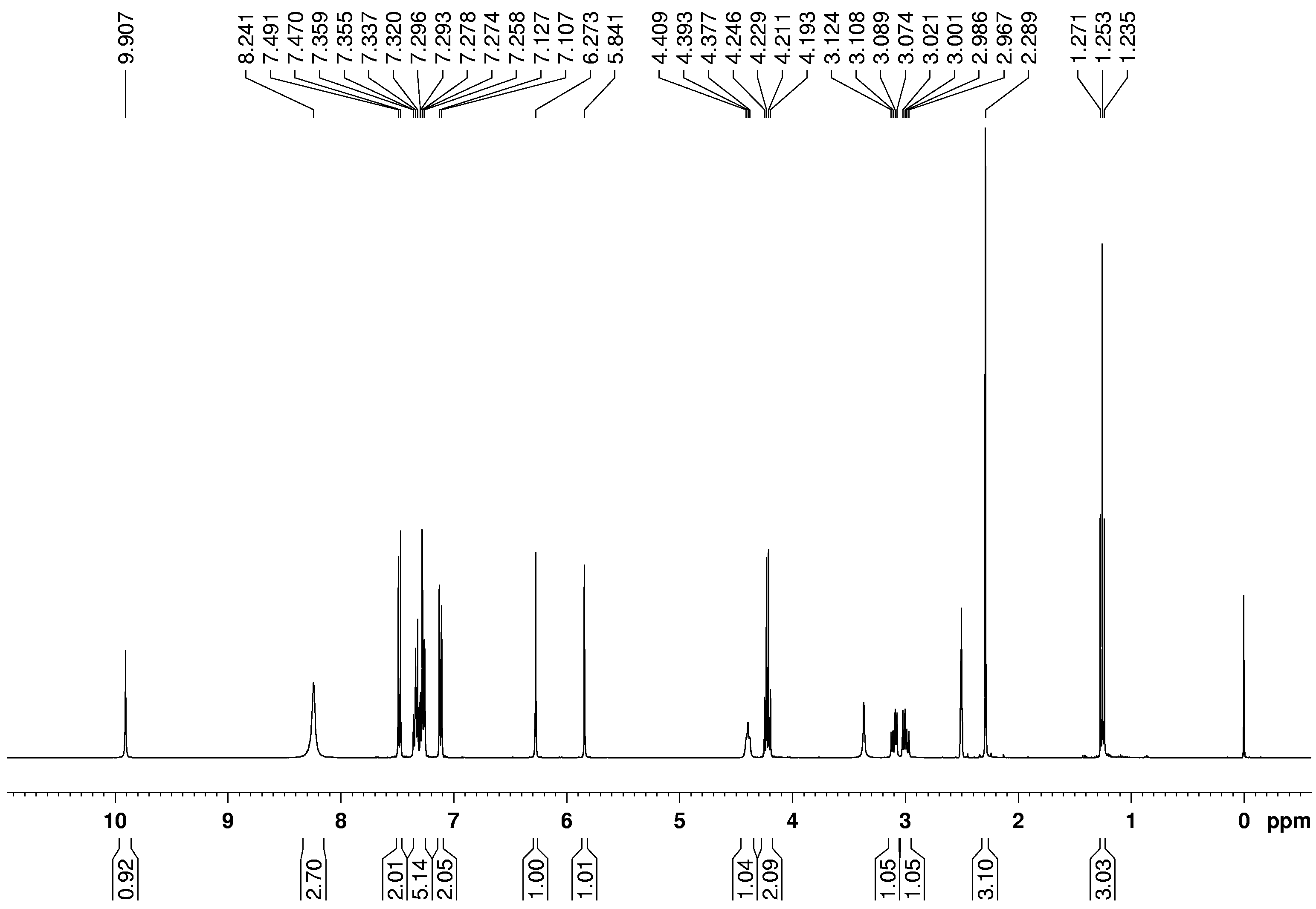


**^13^C NMR spectrum of (S)Phe-ΔAla-OEt·Tos**


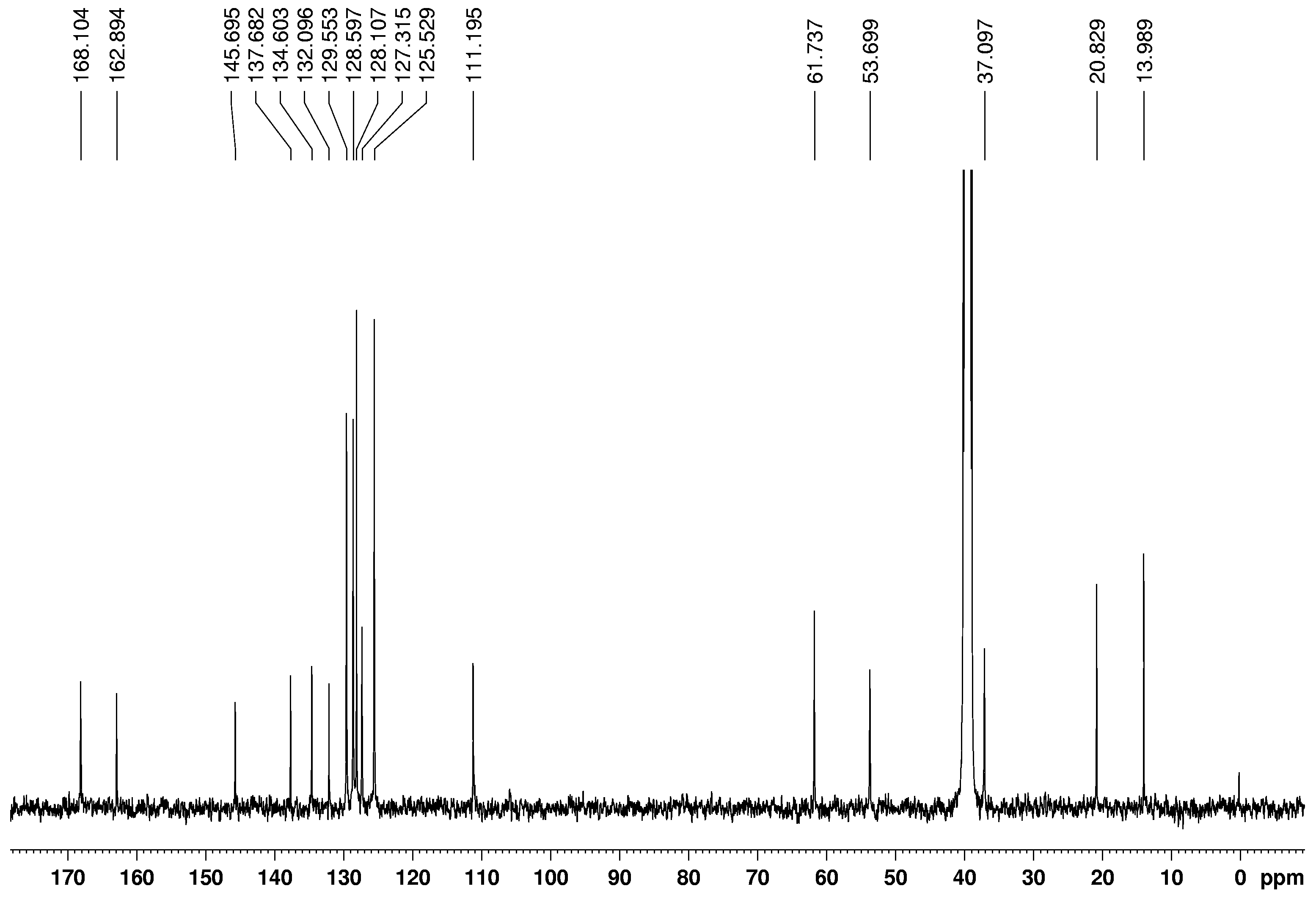


**^1^H NMR spectrum of (S)Phe-ΔAla-OPr^i^·Tos**


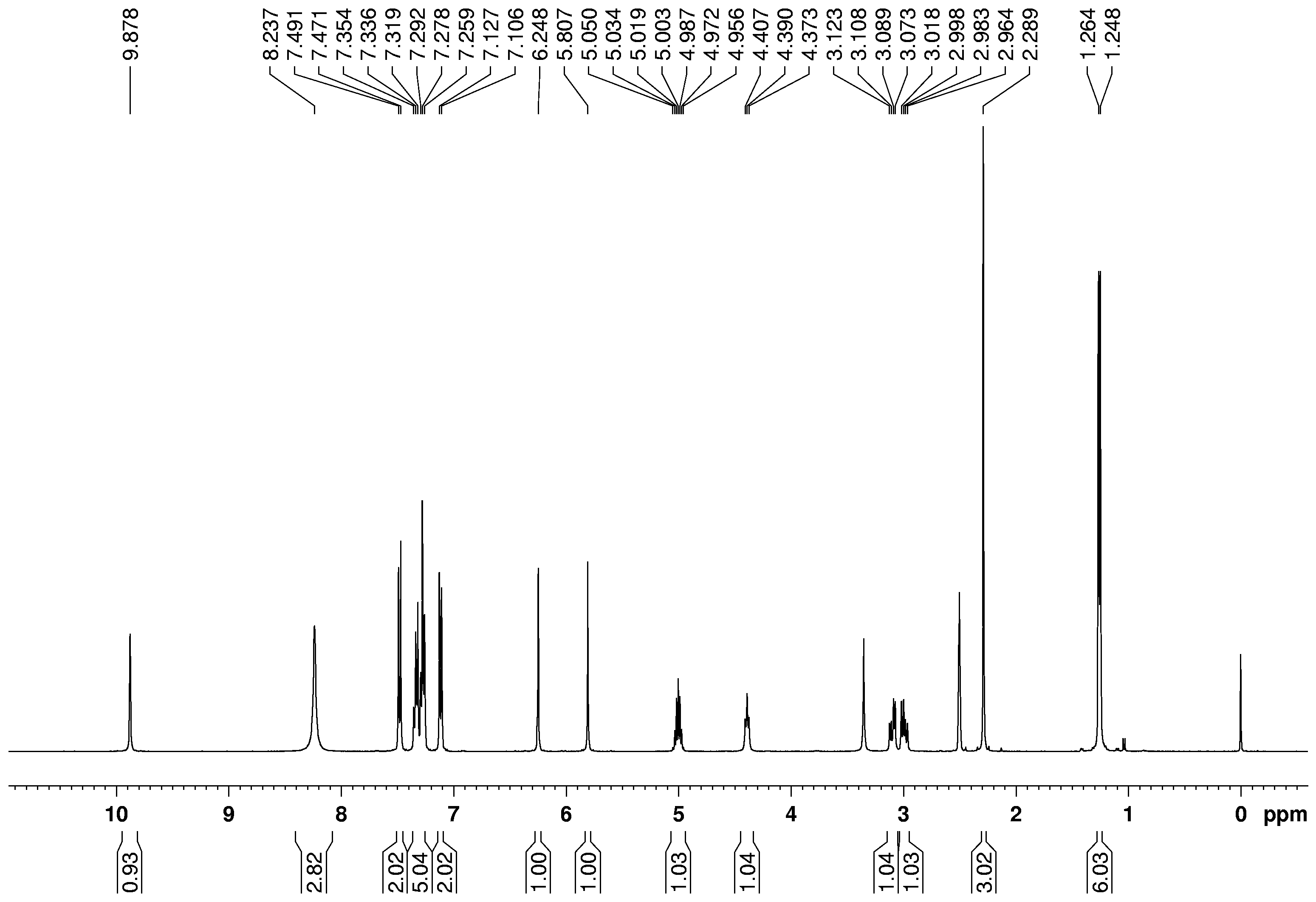


**^13^C NMR spectrum of (S)Phe-ΔAla-OPr^i^·Tos**


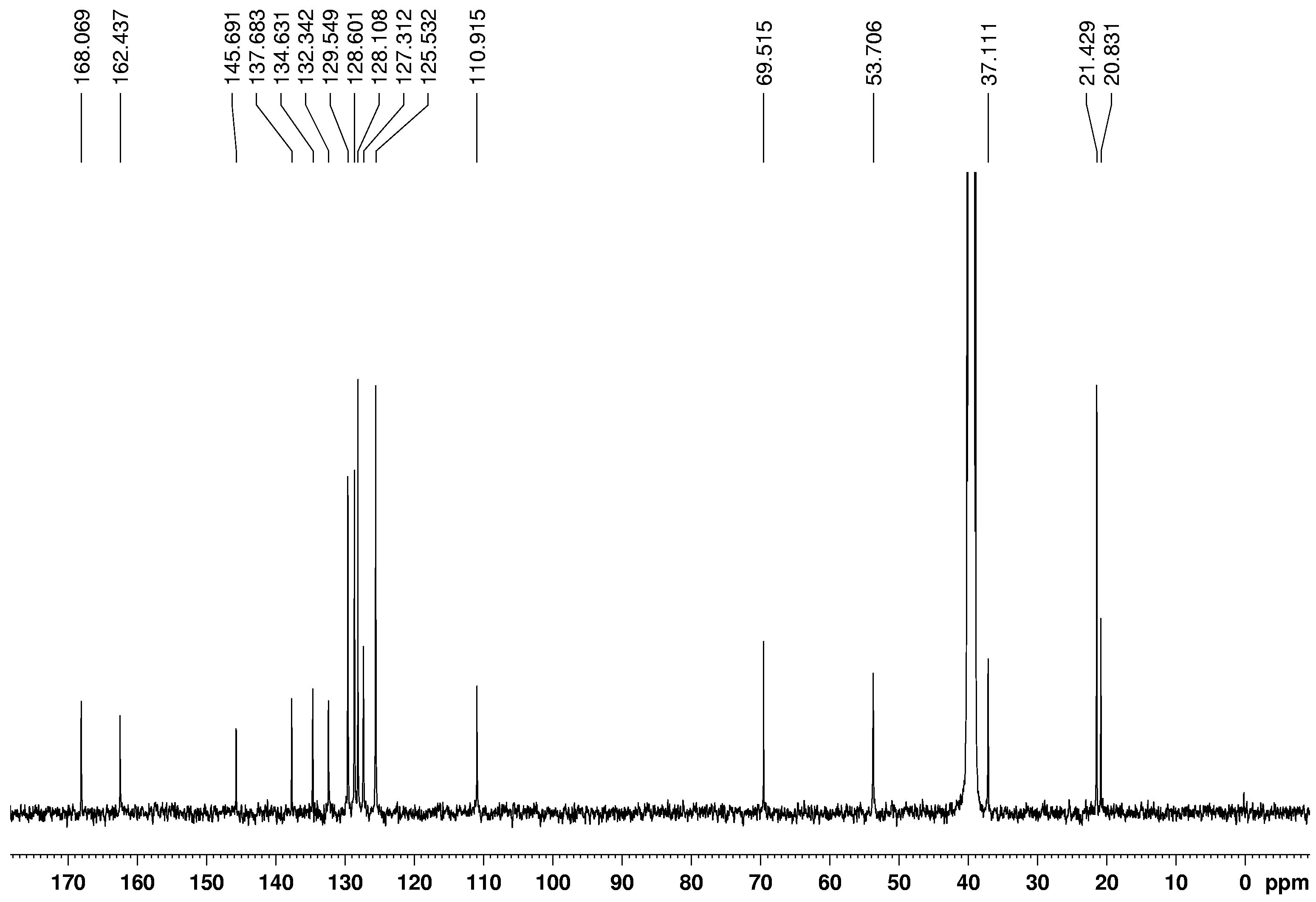


**^1^H NMR spectrum of Gly-ΔAla-OAll·Tos**

***
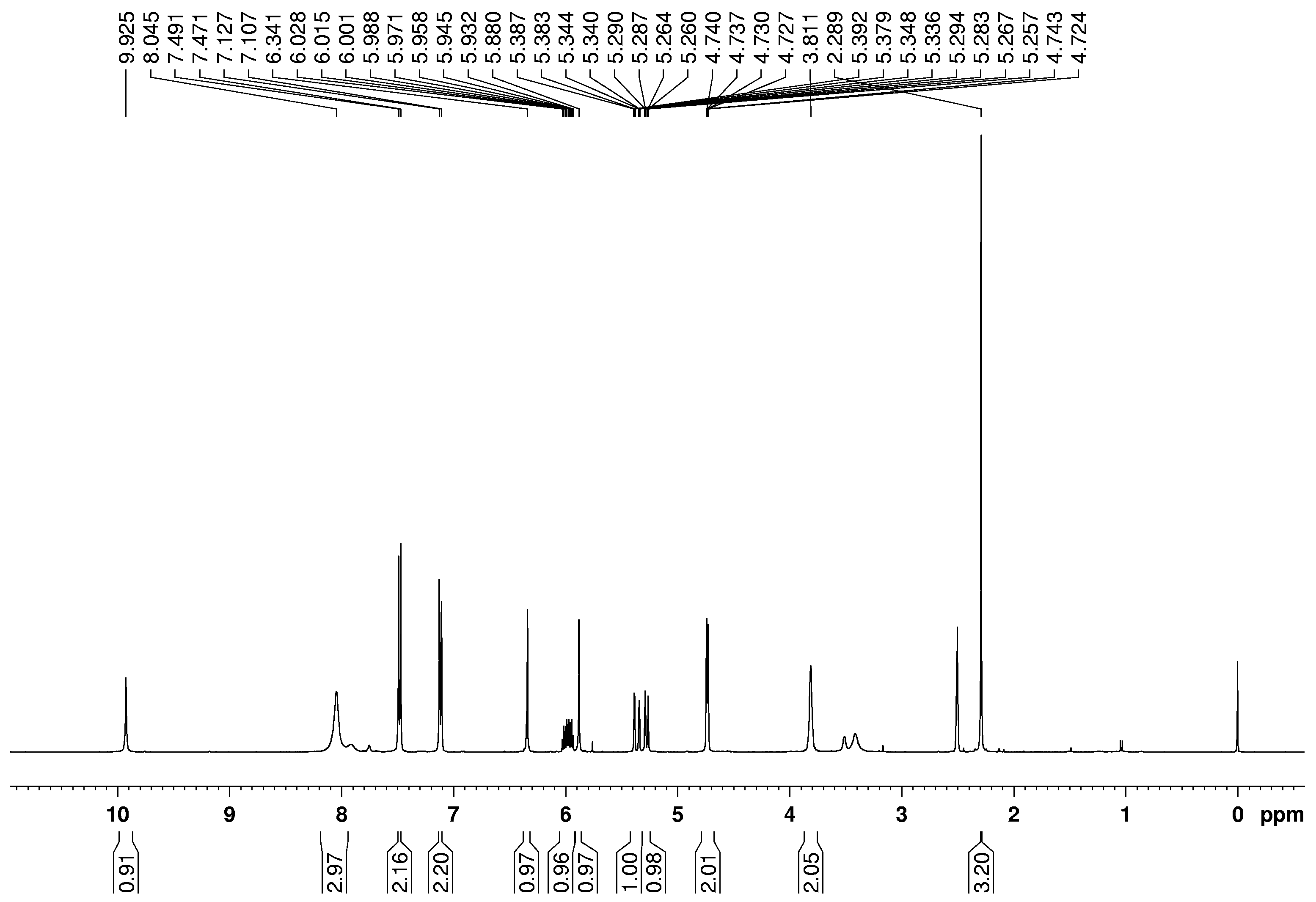
***

**^13^C NMR spectrum of Gly-ΔAla-OAll·Tos**


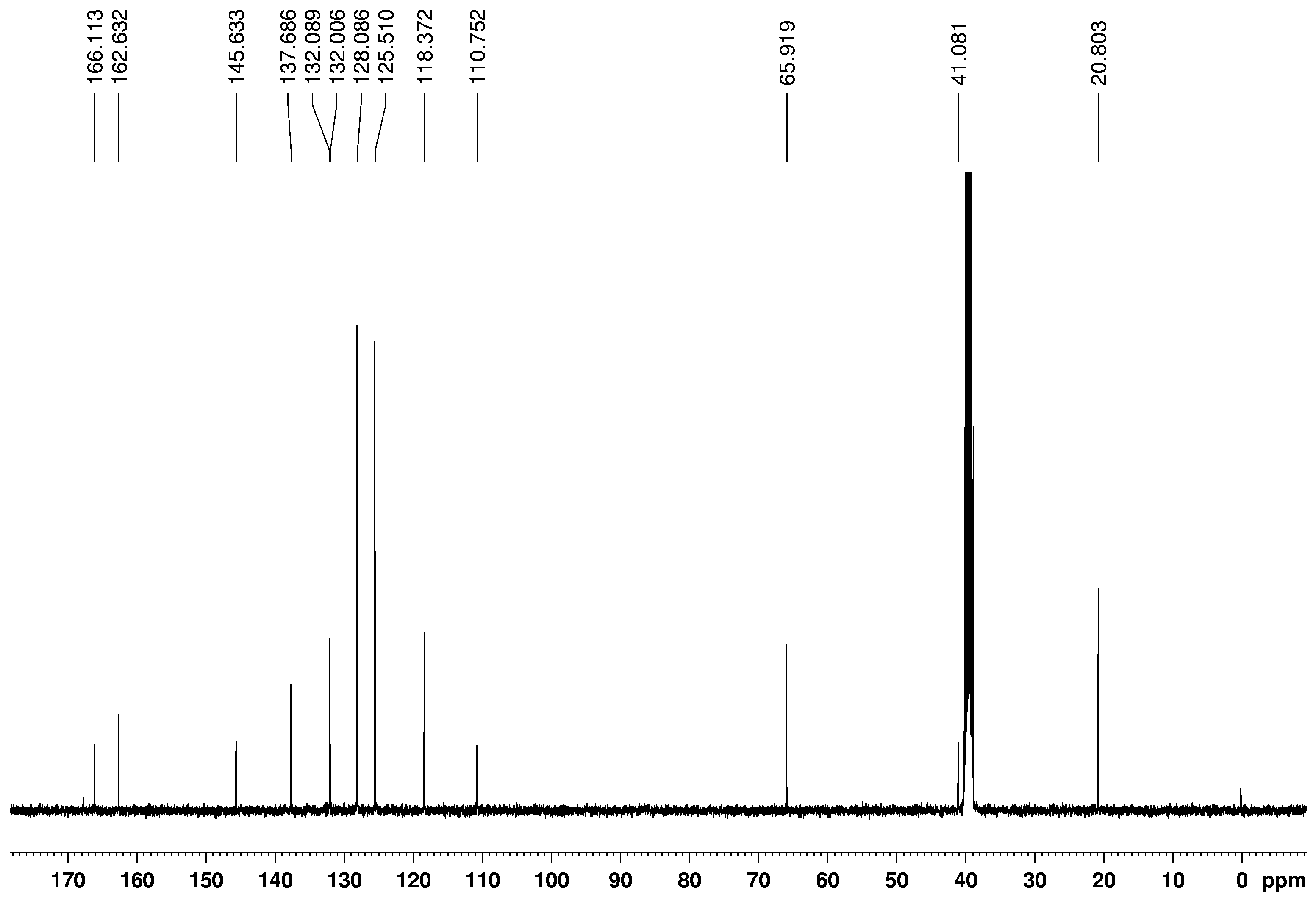


**^1^H NMR spectrum of Gly-ΔAla-OPrg·Tos** (isopropanol is seen as impurity)

**
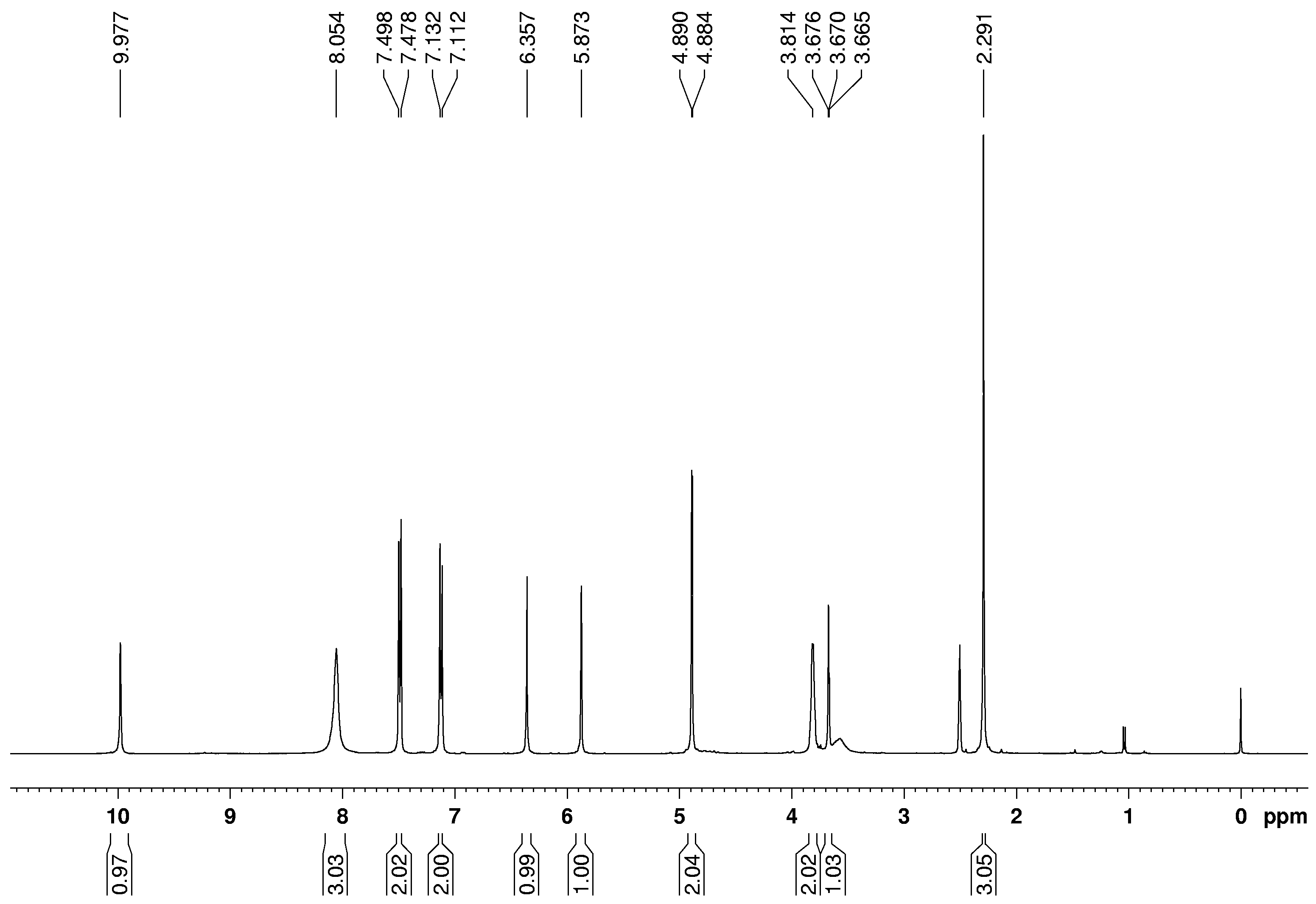
**

**^13^C NMR spectrum of Gly-ΔAla-OPrg·Tos** (isopropanol is seen as impurity)

**
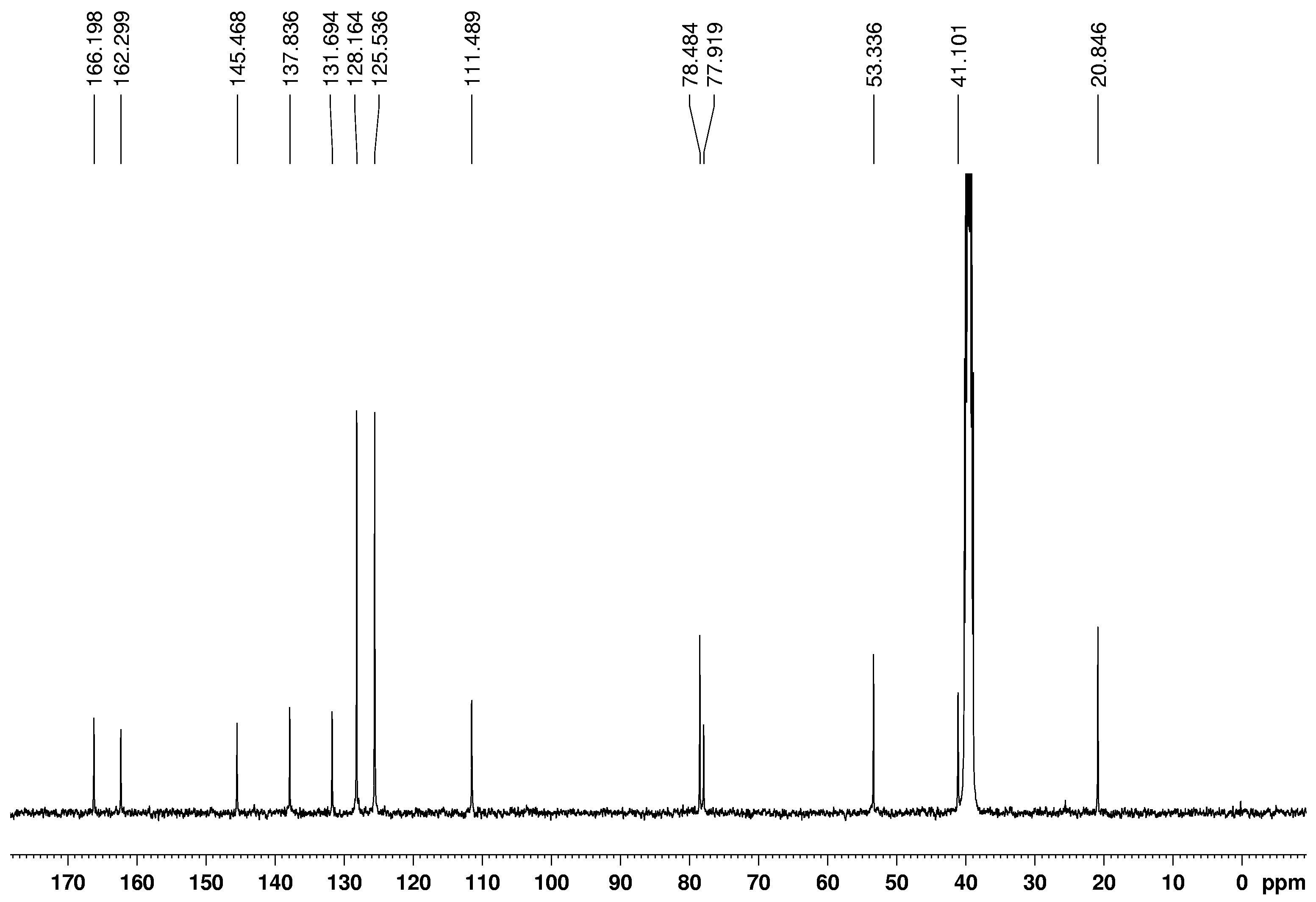
**

**^1^H NMR spectrum of (S)Phe-ΔAla-OAll·Tos** (isopropanol is seen as impurity)


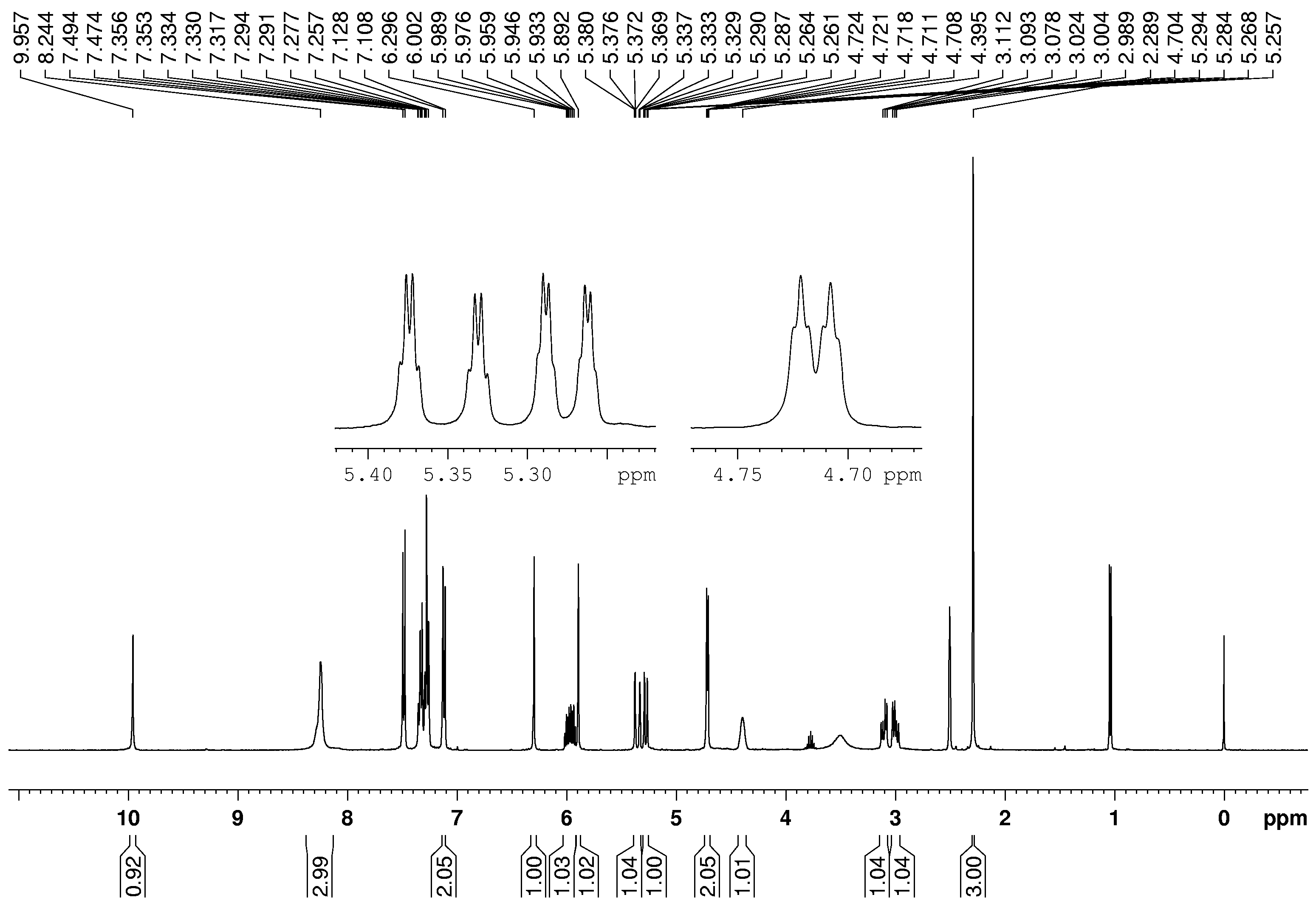


**^13^C NMR spectrum of (S)Phe-ΔAla-OAll·Tos** (isopropanol is seen as impurity)


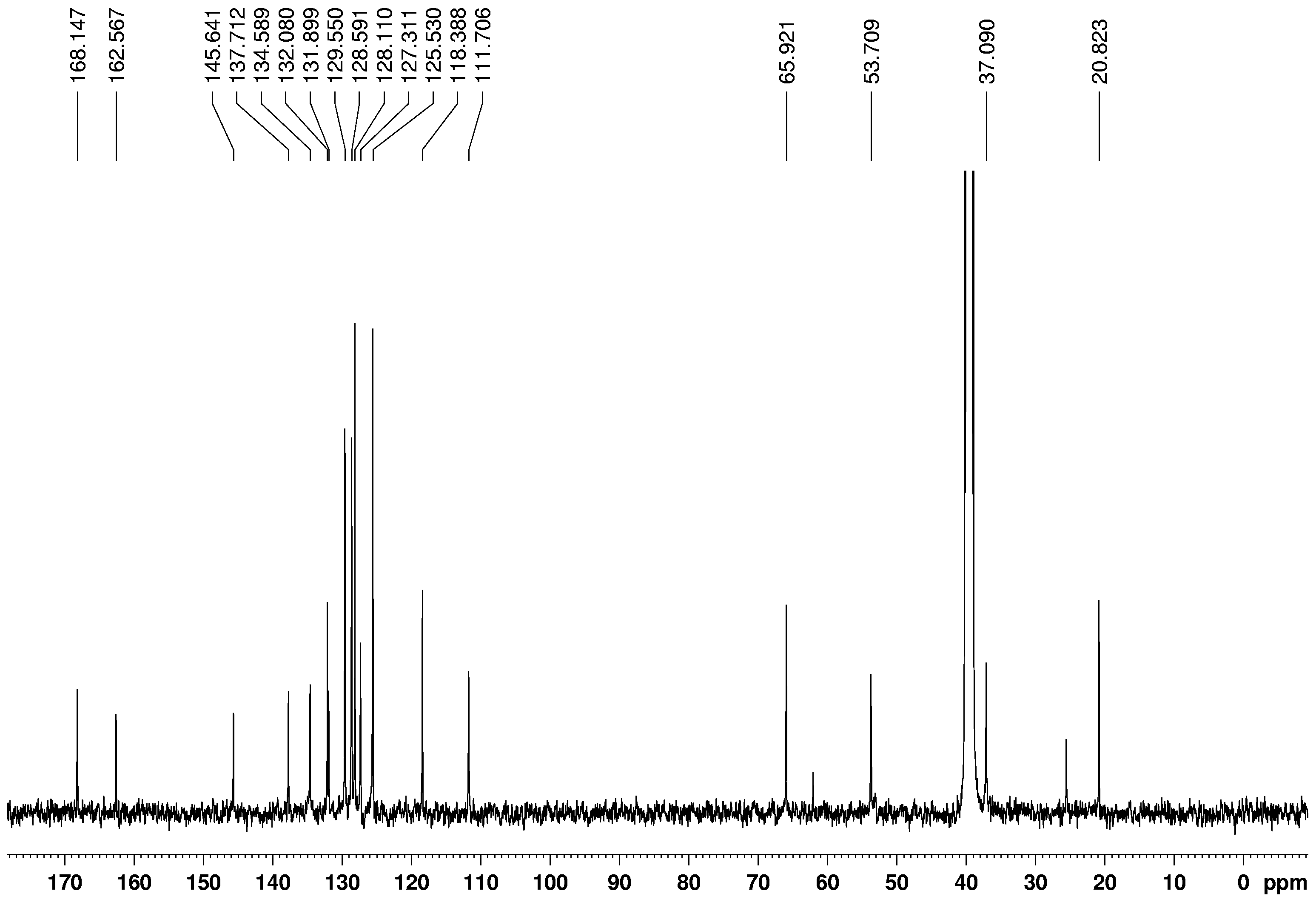


**^1^H NMR spectrum of (S)Phe-ΔAla-OPrg·Tos**

**
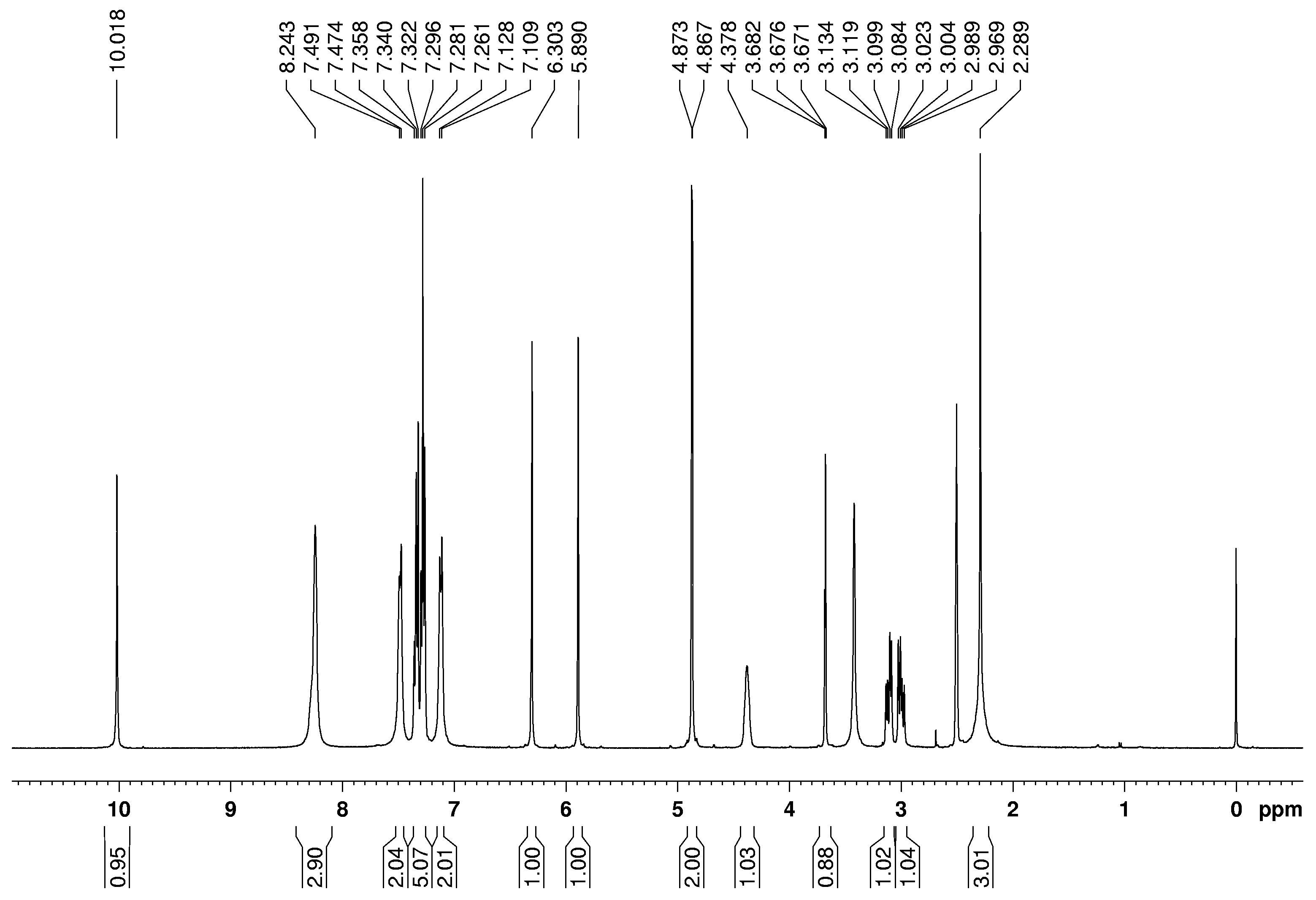
**

**^13^CNMR spectrum of (S)Phe-ΔAla-OPrg·Tos**

***
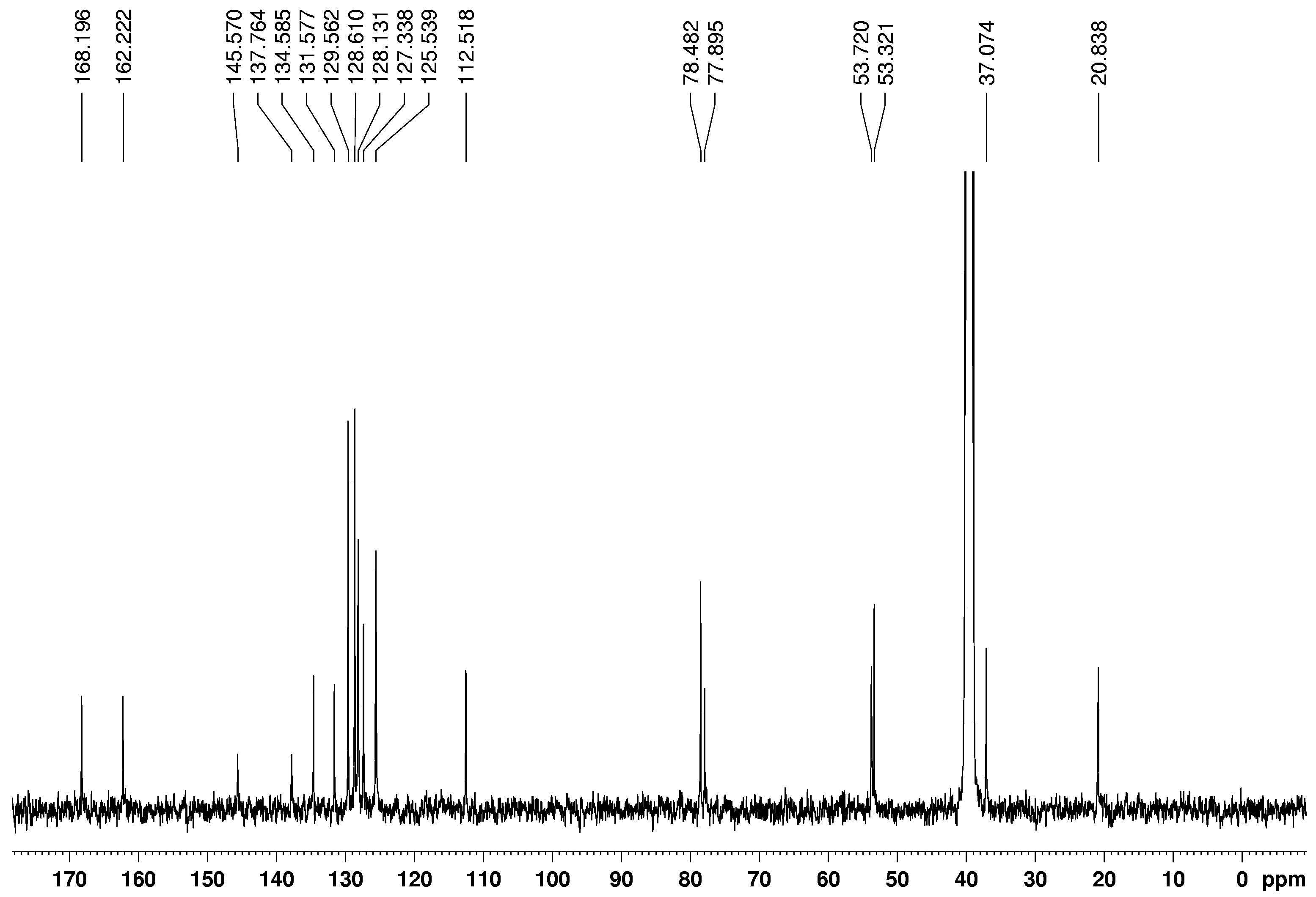
***

**^1^H NMR spectrum of Gly-Δ^Z^Phe-OAll·TFA** (dichloromethane is seen as impurity)


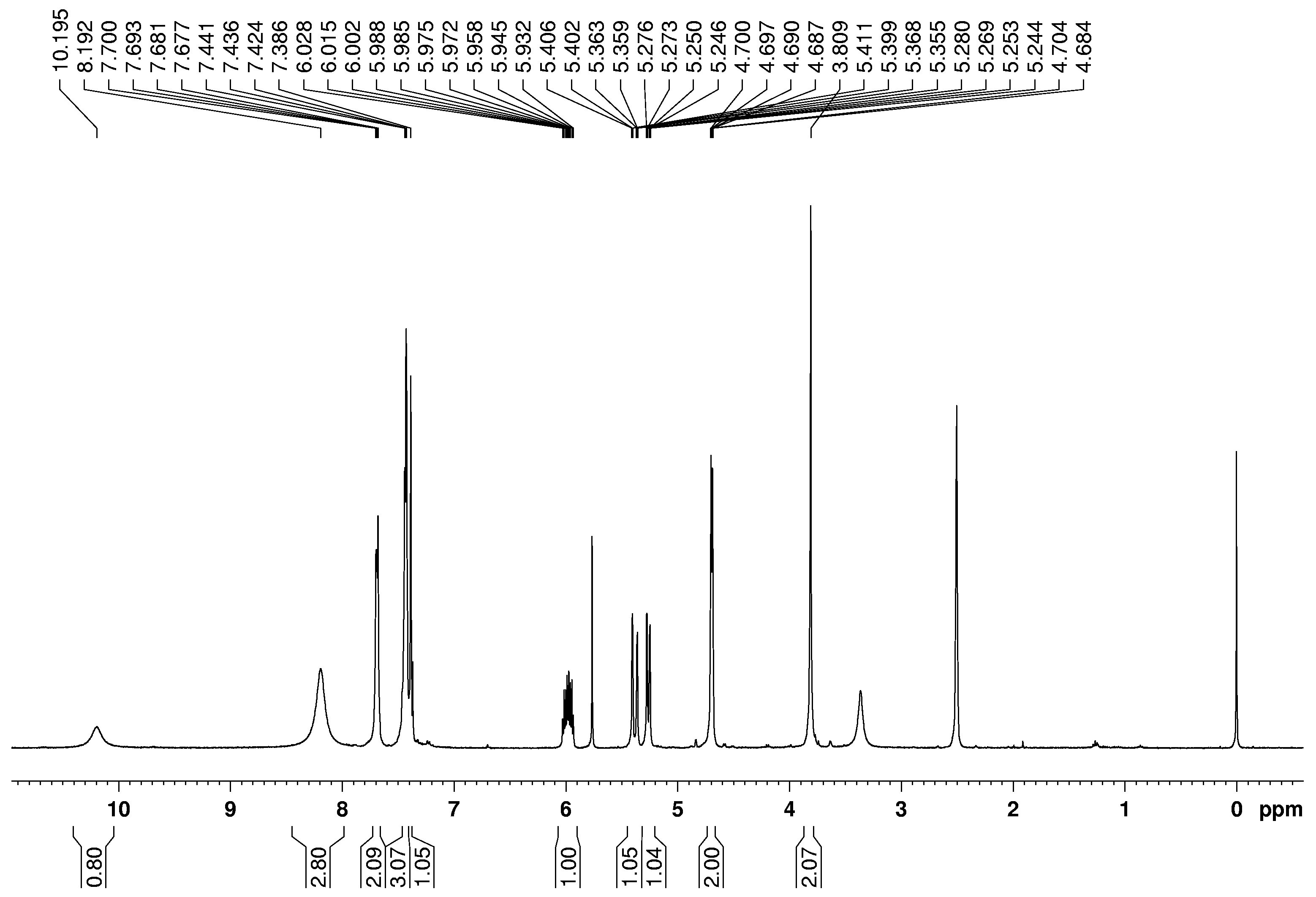


**^13^C NMR spectrum of Gly-Δ^Z^Phe-OAll·TFA** (dichloromethane is seen as impurity)


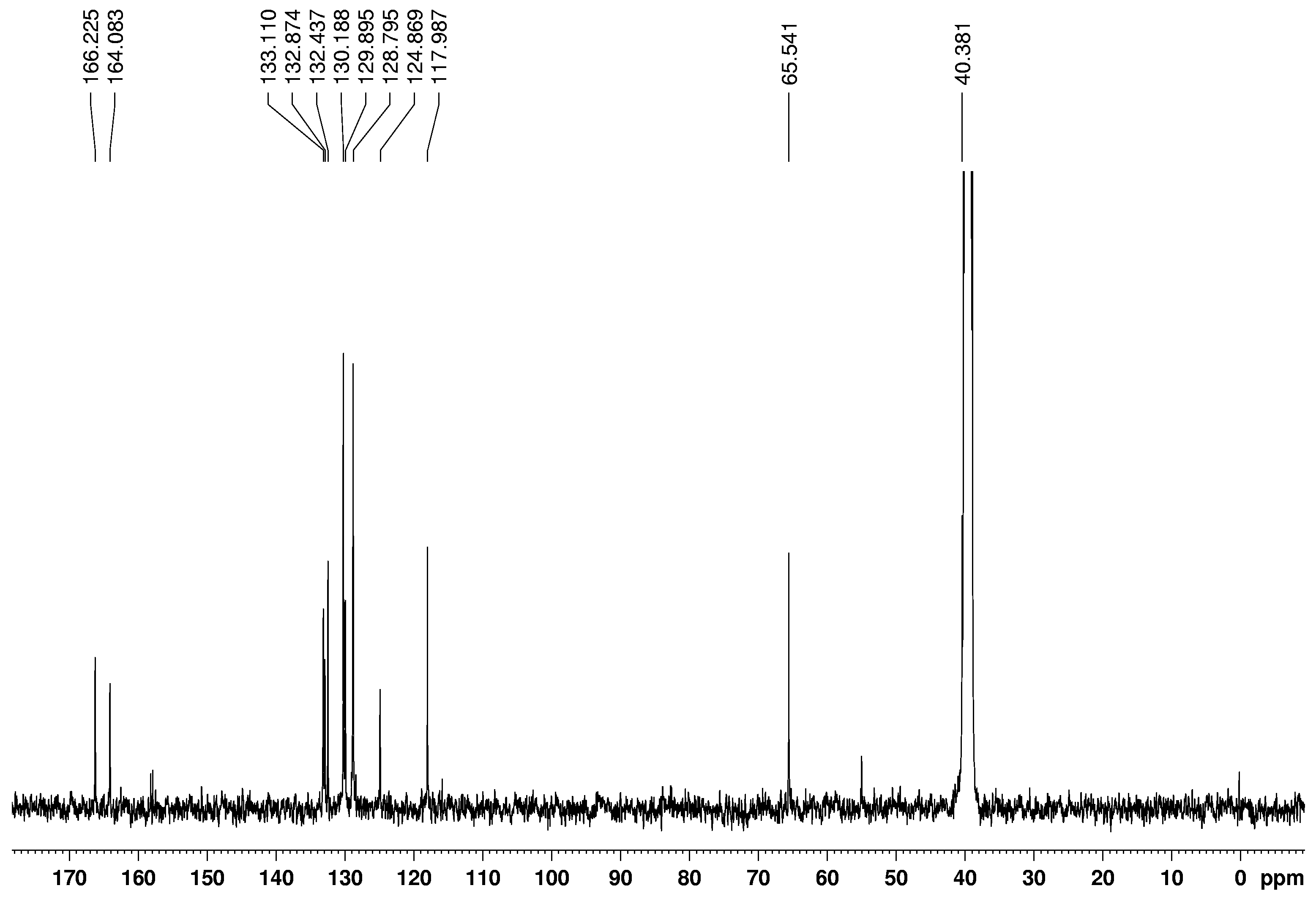


**^1^H NMR spectrum of Gly-Δ^Z^Phe-OPrg·TFA** (dichloromethane is seen as impurity)

**
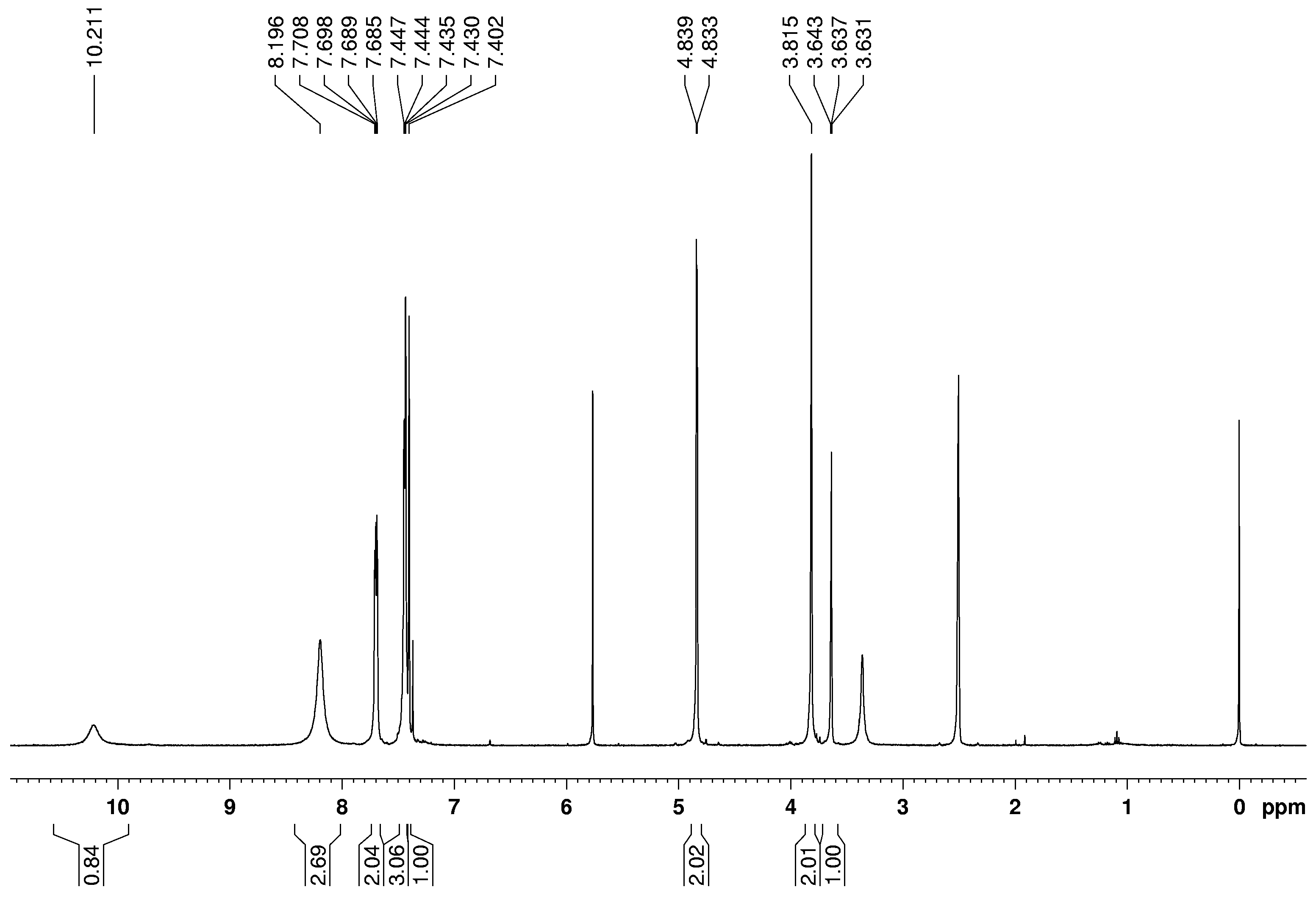
**

**^13^C NMR spectrum of Gly-Δ^Z^Phe-OPrg·TFA** (dichloromethane is seen as impurity)

**
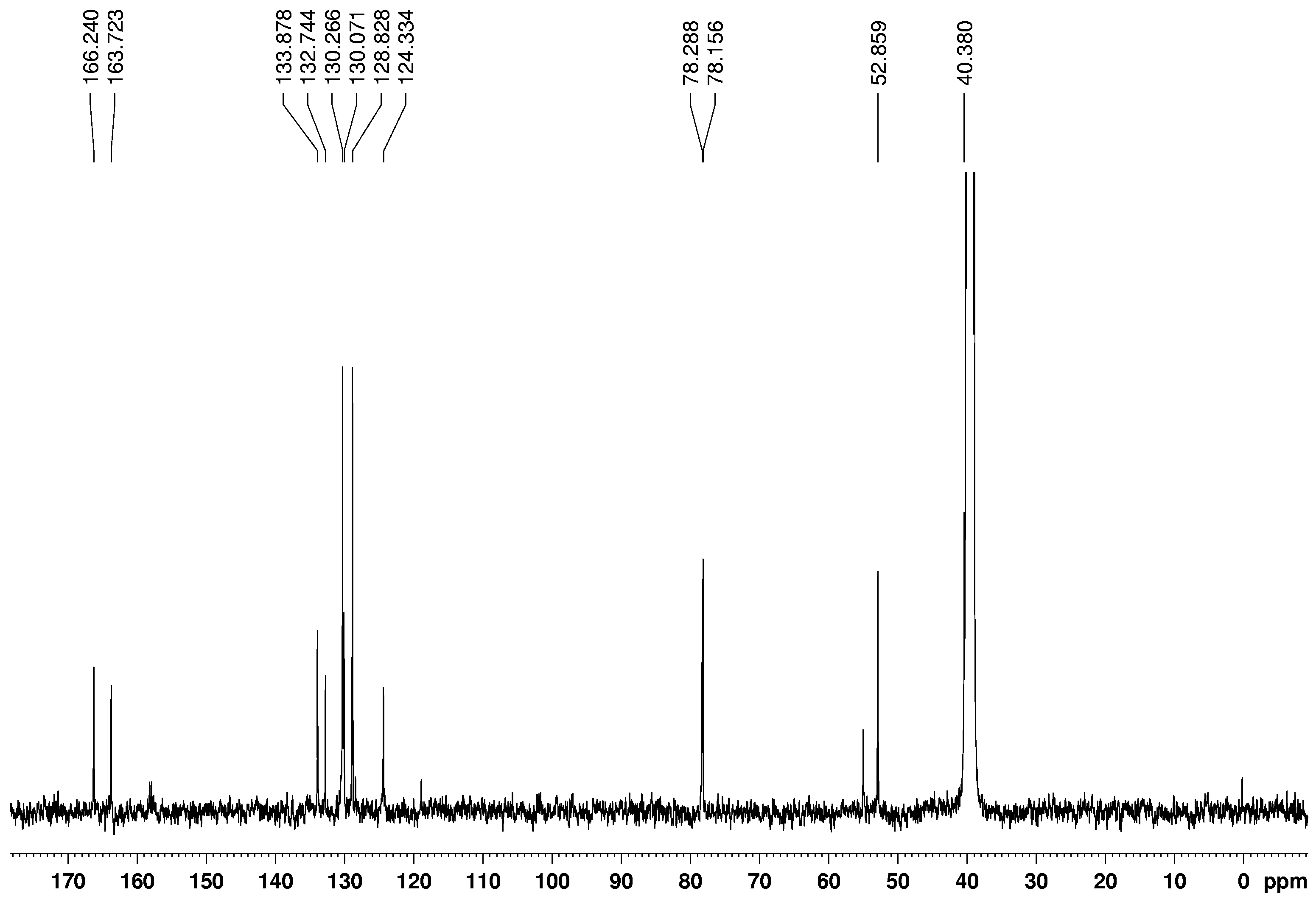
**

**^1^H NMR spectrum of Gly-Δ^Z^Phe-OCH_2_CH(OH)CH_2_Cl·HCl** (isopropanol is seen as impurity)

**
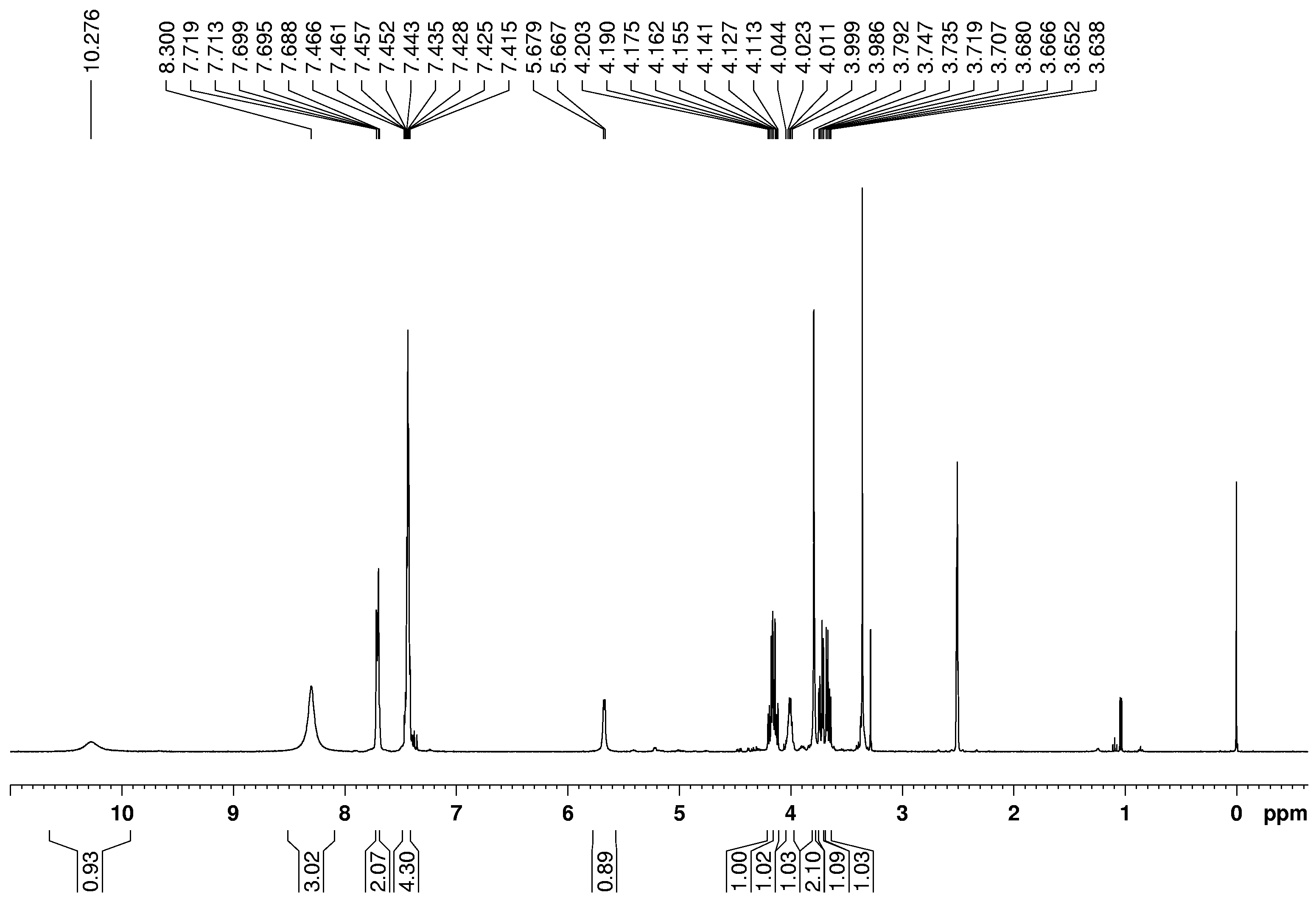
**

**^13^C NMR spectrum of Gly-Δ^Z^Phe-OCH_2_CH(OH)CH_2_Cl·HCl**

**
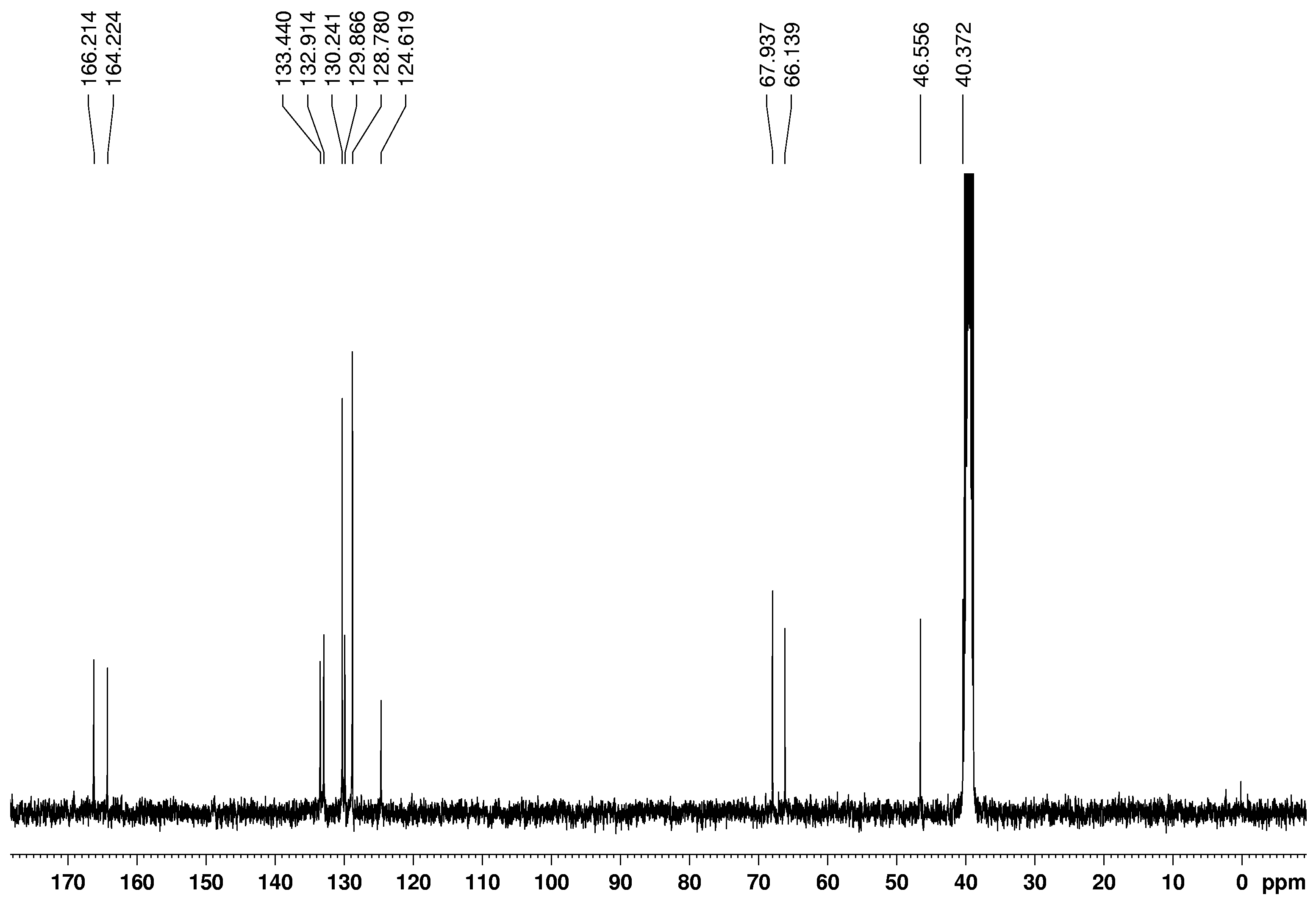
**
